# Differences in IgG Fc Glycosylation Are Associated with Outcome of Pediatric Meningococcal Sepsis

**DOI:** 10.1128/mBio.00546-18

**Published:** 2018-06-19

**Authors:** Noortje de Haan, Navin P. Boeddha, Ebru Ekinci, Karli R. Reiding, Marieke Emonts, Jan A. Hazelzet, Manfred Wuhrer, Gertjan J. Driessen

**Affiliations:** a Center for Proteomics and Metabolomics, Leiden University Medical Center, Leiden, The Netherlands; b Intensive Care and Department of Pediatric Surgery, Erasmus MC, Sophia Children’s Hospital, University Medical Center Rotterdam, Rotterdam, The Netherlands; c Division of Pediatric Infectious Diseases & Immunology, Department of Pediatrics, Erasmus MC-Sophia Children’s Hospital, University Medical Center Rotterdam, Rotterdam, The Netherlands; d Institute of Cellular Medicine, Newcastle University, Newcastle upon Tyne, United Kingdom; e Department of Paediatric Immunology, Infectious Diseases & Allergy, Great North Children’s Hospital, Newcastle upon Tyne Hospitals NHS Foundation Trust, Newcastle upon Tyne, United Kingdom; f NIHR Newcastle Biomedical Research Centre based at Newcastle upon Tyne Hospitals NHS Trust and Newcastle University, Newcastle upon Tyne, United Kingdom; g Department of Public Health, Erasmus University Medical Center Rotterdam, Rotterdam, The Netherlands; h Department of Paediatrics, Juliana Children’s Hospital/Haga Teaching Hospital, The Hague, The Netherlands; Albert Einstein College of Medicine

**Keywords:** children, critical care, Fc glycosylation, immunoglobulin G, meningococcal sepsis, *N*-glycan, outcome

## Abstract

Pediatric meningococcal sepsis often results in morbidity and/or death, especially in young children. Our understanding of the reasons why young children are more susceptible to both the meningococcal infection itself and a more fulminant course of the disease is limited. Immunoglobulin G (IgG) is involved in the adaptive immune response against meningococcal infections, and its effector functions are highly influenced by the glycan structure attached to the fragment crystallizable (Fc) region. It was hypothesized that IgG Fc glycosylation might be related to the susceptibility and severity of meningococcal sepsis. Because of this, the differences in IgG Fc glycosylation between 60 pediatric meningococcal sepsis patients admitted to the pediatric intensive care unit and 46 age-matched healthy controls were investigated, employing liquid chromatography with mass spectrometric detection of tryptic IgG glycopeptides. In addition, Fc glycosylation profiles were compared between patients with a severe outcome (death or the need for amputation) and a nonsevere outcome. Meningococcal sepsis patients under the age of 4 years showed lower IgG1 fucosylation and higher IgG1 bisection than age-matched healthy controls. This might be a direct effect of the disease; however, it can also be a reflection of previous immunologic challenges and/or a higher susceptibility of these children to develop meningococcal sepsis. Within the young patient group, levels of IgG1 hybrid-type glycans and IgG2/3 sialylation per galactose were associated with illness severity and severe outcome. Future studies in larger groups should explore whether IgG Fc glycosylation could be a reliable predictor for meningococcal sepsis outcome.

## IMPORTANCE

Meningococcal sepsis causes significant mortality and morbidity worldwide, especially in young children. Identification of risk factors for a more fulminant infection would help to decide on appropriate treatment strategies for the individual patients. Immunoglobulin G (IgG) plays an essential role in humoral immune responses and is involved in the adaptive immune response against meningococcal infections. Of great influence on the receptor affinity of IgG is the *N*-glycan on its fragment crystallizable (Fc) portion. In the present study, we analyzed IgG glycosylation during the fast development of meningococcal sepsis in children, and we were able to identify glycosylation features that are different between meningococcal sepsis patients and healthy controls. These features might be indicative of a higher susceptibility to meningococcal sepsis. In addition, we found glycosylation features in the patients that were associated with illness severity and severe disease outcome, having the potential to serve as a disease outcome predictor.

## INTRODUCTION

Meningococcal infections continue to cause significant mortality and morbidity, despite important reductions in the number of cases as a result of vaccination programs (1, 2). Several factors associated with susceptibility and/or severity have been identified, e.g., living in crowded conditions, passive smoking and antecedent viral infections (3, 4). In addition, younger age (<4 years) is an important risk factor for both disease susceptibility and severity, presumably due to an immature immune system (1, 5, 6). However, the exact mechanism causing young children to be more vulnerable and the factors determining the course of disease are largely unknown (5). Besides identifying risk factors for the susceptibility of children to be admitted to the pediatric intensive care unit (PICU) with meningococcal sepsis, there is a great interest in identifying prognostic markers to predict the course of the disease and severe outcomes (e.g., death or the need for amputation). These markers would help to decide on appropriate treatment strategies for the individual patients (7). Several laboratory markers have proven to be of predictive value for the course of meningococcal sepsis, including levels of procalcitonin, C-reactive protein (CRP), leukocytes, thrombocytes, plasminogen activator inhibitor 1 (PAI-1), fibrinogen, and various cytokines (8–10). In addition to the individual markers, predictive scores were developed and validated for the course of meningococcal sepsis, among which the pediatric risk of mortality (PRISM) score (11), the Rotterdam score (9), and the base rate and platelet count (BEP) score (12). These scores were all reported to have a good predictive performance for meningococcal sepsis mortality with an area under the concentration-time curve (AUC) between 0.80 and 0.96 (12).

Immunoglobulin G (IgG) plays an essential role in humoral immune responses and is highly involved in the adaptive immune response against meningococcal infections (13, 14). IgG specific for meningococcal serogroup B is able to initiate complement-dependent lysis of the bacterium and leukocyte-mediated phagocytosis (15, 16). Both the complement- and the leukocyte-mediated effector functions are mainly induced by IgG1 and IgG3, while the other two IgG subclasses show less activity (IgG2) or no activity at all (IgG4) (14). It was suggested that the severity of the disease in young children in particular is not determined by the abundance of (certain subclasses of) antimeningococcal IgG, but rather by either the specificity or affinity of the IgG molecule for the antigen or the IgG receptors (13).

Of great influence on the receptor affinity of IgG is the *N*-glycan on its fragment crystallizable (Fc) at Asn297 (17, 18). The Fc portion of the IgG molecule is *N*-glycosylated in the endoplasmic reticulum (ER) and Golgi apparatus of B lymphocytes, a process that is under the regulation of both genetic factors and environmental B cell stimuli (19–21). Functional studies have shown the effect of alterations in IgG Fc glycosylation on the binding affinity to both Fcγ receptors (FcγR) and complement factor C1q (22). For example, increased IgG1 Fc galactosylation showed increased C1q binding and complement-dependent cytotoxicity (CDC) (22, 23). Of note, afucosylation of IgG1 Fc glycans resulted in substantially increased binding of the antibody to FcγRIIIa and FcγRIIIb, which resulted in increased antibody-dependent cellular cytotoxicity (ADCC) (18, 22).

In addition to the influence of IgG Fc glycosylation on receptor interaction, changes in the glycosylation are also associated with various inflammatory diseases, like active tuberculosis infections (24), HIV (25), alloimmune cytopenias (26, 27), and autoimmune diseases like rheumatoid arthritis (28) and inflammatory bowel disease (29, 30). Because of the large influence of IgG glycosylation on antibody function and its association with inflammatory processes, we hypothesize that the fast development of meningococcal sepsis could be associated with Fc glycosylation profiles in the total plasma IgG pool. The aim of this study was to identify differences in IgG Fc glycosylation between pediatric meningococcal sepsis patients and age-matched healthy controls. In addition, we evaluated the potential of specific glycosylation features to serve as a predictive marker for disease outcome.

## RESULTS

**FIG 1:**
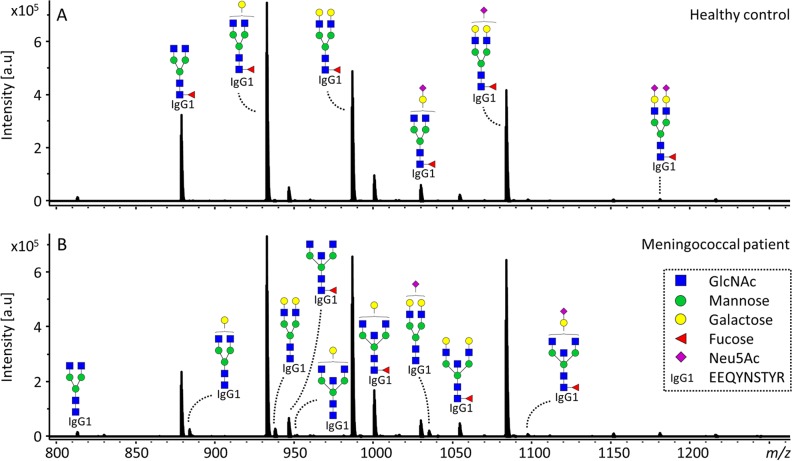
IgG1 glycoforms detected in healthy controls and meningococcal patients. (A and B) Representative mass spectra of a healthy 2.8-year-old boy (A) and a 2.8-year-old male meningococcal patient (B). Annotated are the 15 overall most abundant IgG1 glycoforms; the glycoforms that were found to be higher in the meningococcal patients compared to healthy controls (diantennary glycans without fucose or with a bisecting GlcNAc) are indicated in the spectrum of the patient (B). The proposed glycan structures are based on fragmentation and literature (20, 31). Green circles, mannose; yellow circles, galactose; blue squares, *N*-acetylglucosamine (GlcNAc); red triangles, fucose; pink diamonds, *N*-acetylneuraminic acid (Neu5Ac). a.u., arbitrary units.

IgG Fc glycosylation was analyzed in a subclass-specific way for 60 pediatric patients with a meningococcal infection admitted to the PICU, as well as for 46 age- and sex-matched healthy controls (Table 1; see Table S1 in the supplemental material). For all samples, 22 IgG1 Fc glycoforms were quantified, for 57 cases and 34 controls, 15 IgG2/3 glycoforms were quantified, and for 48 cases and 29 controls, 10 IgG4 glycoforms were quantified (Fig. 1; see Tables S1 and S2 in the supplemental material). From these directly measured glycan traits, derived traits were calculated per IgG subclass. This was done based on glycan type (complex or hybrid-type), bisection, fucosylation, galactosylation, and sialylation (Table 2 and Table S2). The samples of the healthy controls, remeasured in the present study, were a subset of a larger cohort previously analyzed using a different technique, based on the matrix-assisted laser desorption ionization–time-of-flight mass spectrometry (MALDI-TOF MS) detection of derivatized glycopeptides (31). The healthy controls featured a lower IgG1 fucosylation and IgG1 and IgG2/3 sialylation with higher age, as already previously reported for this control cohort (see Fig. S1 and Table S3 in the supplemental material). In addition, we detected a lower relative abundance of IgG1 and IgG2/3 hybrid-type glycans with higher age in the healthy controls, as well as a higher abundance of bisected glycans on IgG2/3 with higher age, both not previously described for healthy children in this age category (Fig. S1) (31, 32).

10.1128/mBio.00546-18.2 FIG S1  Correlation between IgG Fc glycosylation and age (years) in the healthy controls. The Spearman’s correlation coefficient is represented in red for a positive correlation and in blue for a negative correlation between glycan trait and age (A). Periods indicate *P* < 0.05, and crosses indicate *P* < 2.7 × 10^−3^ (α = 2.7 × 10^−3^ after 5% FDR correction). Newly reported glycan correlations with age in healthy children were the negative association of levels of IgG1 and IgG2/3 hybrid-type glycans with age (B and C) and the positive association of IgG2/3 bisection with age (D). The number of samples for which subclass-specific glycosylation data were available can be found in Table S1. Download FIG S1, TIF file, 0.5 MB. Copyright © 2018 de Haan et al. 2018 de Haan et al. This content is distributed under the terms of the Creative Commons Attribution 4.0 International license.

10.1128/mBio.00546-18.6 TABLE S1  Sample numbers. The number of patients for whom specific clinical data were present is shown in the upper part of the table. Numbers are presented for the whole data set as well as for the different age subgroups (below and above 4 years). The number of samples for which subclass-specific glycosylation data are present is shown in the bottom part of the table. Data were excluded based on spectrum quality or after outlier removal, as described in Materials and Methods. Download TABLE S1, PDF file, 0.3 MB. Copyright © 2018 de Haan et al. 2018 de Haan et al. This content is distributed under the terms of the Creative Commons Attribution 4.0 International license.

10.1128/mBio.00546-18.7 TABLE S2  Direct and derived glycosylation traits. In the upper part of the table, the theoretical mass of the triply charged glycopeptide is shown when the corresponding glycoform was detected for the specific IgG subclass. The individual glycoforms were grouped based on their glycosylation features as described before for IgG glycopeptides in humans (31) and shown in the lower part of the table. The depictions of the derived traits show the minimally required composition to contribute to a trait. H, hexose; N, *N*-acetylhexosamine; F, fucose; S, *N*-acetylneuraminic acid. Green circles, mannose; yellow circles, galactose; blue squares, *N*-acetylglucosamine; red triangles, fucose; pink diamonds, *N*-acetylneuraminic acid. Download TABLE S2, PDF file, 0.5 MB. Copyright © 2018 de Haan et al. 2018 de Haan et al. This content is distributed under the terms of the Creative Commons Attribution 4.0 International license.

10.1128/mBio.00546-18.8 TABLE S3  Correlation between IgG Fc glycosylation and age in healthy children. Spearman’s correlation tests were performed, after multiple-testing correction, *P* values below 2.7 × 10^−3^ were considered statistical significant (indicated in boldface). Download TABLE S3, PDF file, 0.2 MB. Copyright © 2018 de Haan et al. 2018 de Haan et al. This content is distributed under the terms of the Creative Commons Attribution 4.0 International license.

**TABLE 1:** Baseline characteristics of children admitted to the PICU with meningococcal sepsis and of their age-matched healthy controls^a^

Parameter	Result for patients or controls:		
	All	<4 yr old	≥4 yr old
Patients			
*n*	60	37	22
Age, yr (IQR)	2.5 (1.5–8.8)	1.8 (1.2–2.4)	10.1 (6.7–12.3)
Sex, male, *n* (%)	35 (59)	23 (62)	12 (55)
Illness severity			
PRISM score (IQR)	20 (12–25)	21 (14–25)	19 (9–27)
*P* (death Rotterdam)	11 (1–82)	32 (2–89)	5 (1–14)
DIC score (IQR)	5 (4–6)	5 (4–7)	5 (4–7)
Coagulation markers			
Thrombocytes, ×10^6^/liter (IQR)	97 (54–150)	92 (49–166)	109 (82–138)
Fibrinogen, g/liter (IQR)	2.3 (0.9–3.2)	2.3 (0.9–4.0)	2.2 (1.1–2.9)
PAI-1, µg/ml (IQR)	4.8 (2.7–6.9)	5.4 (3.6–10.7)	4.3 (1.5–6.0)
Inflammatory markers			
Leukocytes, ×10^9^/liter (IQR)	7.8 (4.0–15.3)	7.1 (3.4–14.3)	11.0 (5.5–17.2)
CRP, mg/liter (IQR)	74 (44–119)	60 (39–115)	91 (69–128)
Procalcitonin, ng/ml (IQR)	281 (83–482)	361 (145–498)	243 (20–468)
TNF-α, pg/ml (IQR)	8.4 (5.0–19.8)	12.0 (5.3–23.0)	5.0 (5.0–17.5)
IL-6, ng/ml (IQR)	72 (18–383)	176 (42–723)	38 (1–258)
IL-8, ng/ml (IQR)	20 (4–119)	33 (5–219)	9 (1–58)
Outcome			
Mortality, *n* (%)	12 (20)	10 (27)	2 (9)
Amputation, *n* (%)	7 (12)	2 (5)	5 (23)
Severe, *n* (%)	19 (32)	12 (32)	7 (32)
Controls			
*n*	46	24	22
Age, yr (IQR)	3.9 (1.4–10.0)	1.5 (0.8–2.8)	10.0 (6.7–11.6)
Sex, male, *n* (%)	28 (61)	15 (63)	13 (59)

a Medians and interquartile ranges are presented, unless indicated differently. PRISM, pediatric risk of mortality (11); *P* (death Rotterdam): predicted death rate based on the Rotterdam score (9); DIC, disseminated intravascular coagulation (50), PAI-1, plasminogen activator inhibitor-1; TNF-α, tumor necrosis factor alpha. The number of samples for which specific clinical data were available can be found in Table S1.

**TABLE 2:**
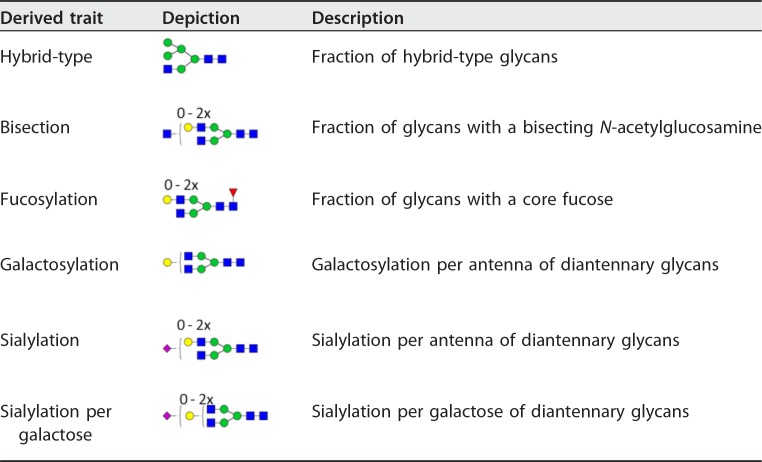
Derived glycosylation traits^a^

a The individual glycoforms were grouped based on their glycosylation features as described before for IgG glycopeptides (31). Green circles, mannose; yellow circles, galactose; blue squares, *N*-acetylglucosamine; red triangles, fucose; pink diamonds, *N*-acetylneuraminic acid. The depictions of the derived traits show the minimally required composition to contribute to the given trait. For detailed calculations of the traits, see Table S2.

### IgG Fc glycosylation differences between patients with meningococcal sepsis and healthy controls.

**FIG 2:**
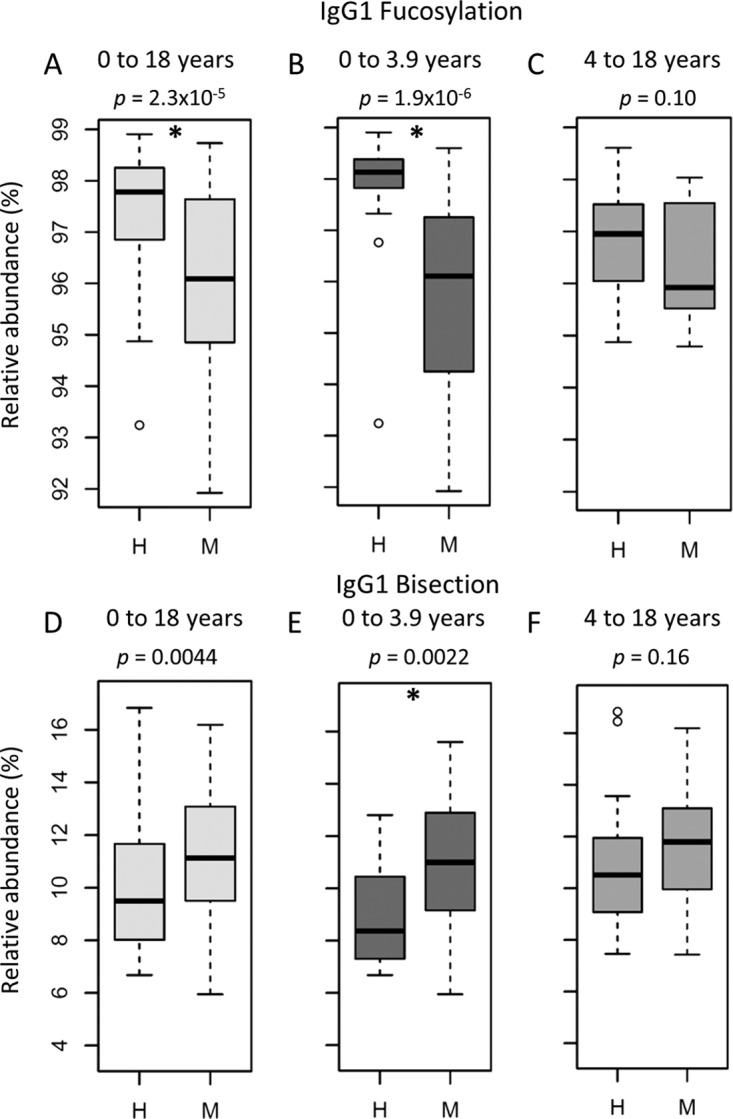
IgG1 Fc fucosylation and bisection are different between meningococcal patients (M) and healthy controls (H) below the age of 4 years. IgG1 Fc fucosylation in meningococcal patients between 0 and 18 (A), 0 and 3.9 (B), and 4 and 18 (C) years old compared to age-matched healthy controls and IgG1 Fc bisection in meningococcal patients between 0 and 18 (D), 0 and 3.9 (E), and 4 and 18 (F) years old compared to age-matched healthy controls. Shown are box and whisker plots, where the boxes represent the interquartile range (IQR) and the whiskers 1.5× IQR. After multiple-testing correction, *P* values below 2.7 × 10^−3^ were considered statistically significant (indicated by an asterisk). The number of samples for which subclass-specific glycosylation data were available can be found in Table S1.

Comparison of the derived glycosylation traits for all IgG subclasses between the meningococcal patients and the healthy controls (see Table S4 in the supplemental material) revealed a lower IgG1 fucosylation in the children with a meningococcal infection (median cases, 96.1%; controls, 97.8%) (Fig. 2A). When comparing children in the younger age category (<4 years old) separately from the older children (≥4 years old), this effect appeared to be strongly present in the younger children (cases, 96.1%; controls, 98.1%) (Fig. 2B and Table 3), while for the older children the difference in IgG1 fucosylation was only detected as a trend (Fig. 2C). Also IgG1 bisection was shown to associate with disease in children below 4, being higher in meningococcal patients (11.0%) than in healthy controls (8.4%) (Fig. 2E and Table 3). In the older age group, a corresponding trend was observed (Fig. 2D and F).

10.1128/mBio.00546-18.9 TABLE S4  Glycosylation differences between pediatric meningococcal patients and age- and sex-matched healthy controls. Mann-Whitney *U* tests were performed to compare the groups. After multiple-testing correction, *P* values below 2.7 × 10^−3^ were considered statistically significant (indicated in boldface). Download TABLE S4, PDF file, 0.3 MB. Copyright © 2018 de Haan et al. 2018 de Haan et al. This content is distributed under the terms of the Creative Commons Attribution 4.0 International license.

**TABLE 3:** Glycosylation differences between pediatric meningococcal patients (0 to 4 years of age) and age- and sex-matched healthy controls and between meningococcal patients with severe and nonsevere disease outcomes^a^

Derived trait	Cases and controls <4 yr old			Meningococcal patients <4 yr old		
	Median % (IQR)		*P* value	Median % (IQR)		*P* value
	Healthy controls	Patients		Nonsevere	Severe	
IgG1						
Hybrid-type	0.45 (0.43–0.52)	0.45 (0.41–0.53)	8.0E−01	**0.50 (0.44–0.54)**	**0.37 (0.35–0.43)**	**2.1E−03**
Bisection	**8.4 (7.4–10.3)**	**11.0 (9.2–12.9)**	**2.0E−03**	10.0 (9.1–11.8)	13.1 (11.6–14.3)	3.0E−02
Fucosylation	**98.1 (97.8–98.4)**	**96.1 (94.2–97.3)**	**1.9E−06**	96.4 (94.6–97.8)	94.5 (93.9–95.8)	2.7E−02
Galactosylation	61.9 (56.7–63.6)	61.6 (58.4–63.3)	8.2E−01	62.2 (59.1–63.4)	59.7 (56.9–62.1)	2.1E−01
Sialylation	11.3 (10.1–12.8)	10.6 (9.7–11.9)	1.5E−01	11.2 (10.3–11.9)	9.6 (9.2–10.4)	1.8E−02
Sialylation per galactose	18.4 (17.7–19.7)	17.8 (16.9–18.7)	3.7E−02	18.2 (17.5–19)	17.2 (15.9–17.6)	1.6E−02
IgG2/3						
Hybrid-type	0.43 (0.40–0.54)	0.39 (0.30–0.49)	9.5E−02	0.43 (0.39–0.51)	0.30 (0.25–0.35)	4.8E−03
Bisection	7.4 (6.3–8.1)	9.7 (8.8–11.2)	4.0E−03	9.2 (8.5–10)	11.1 (9.2–11.6)	1.2E−01
Fucosylation	98.4 (98.3–98.7)	97.7 (97.5–98.1)	5.7E−03	97.8 (97.5–98.4)	97.5 (97.5–98)	4.6E−01
Galactosylation	54.5 (46.7–58.1)	51.4 (49.2–53.6)	5.1E−01	51.8 (49.3–54.4)	51.3 (49.8–52.6)	6.1E−01
Sialylation	13.1 (9.3–14.3)	10.6 (9.7–11.7)	2.1E−01	11.0 (10.5–11.9)	9.6 (9.4–10.3)	8.3E−03
Sialylation per galactose	23.7 (21.4–24.7)	21.1 (19.5–22.6)	1.4E−02	**21.9 (20.5–23.1)**	**19.3 (18.5–20.9)**	**2.0E−03**
IgG4						
Bisection	13.2 (10.6–14.5)	15.5 (13.5–16)	1.7E−01	14.2 (12.4–15.8)	15.9 (15.7–16.1)	3.8E−02
Galactosylation	51.6 (44.5–54.9)	54.5 (52.5–58.2)	7.0E−02	55.8 (52.4–59.1)	53.7 (52.7–55.4)	4.5E−01
Sialylation	14.1 (11.3–14.9)	13.6 (12.4–14.7)	8.5E−01	14.4 (13.4–15.4)	12.5 (12.3–13)	2.3E−02
Sialylation per galactose	26.5 (25.2–28.6)	24.9 (23–26.7)	1.5E−01	25.7 (23.6–27.3)	22.9 (22.6–25.1)	2.7E−02

a Mann-Whitney *U* tests were performed to compare the groups. After multiple-testing correction, *P* values below 2.7 × 10^−3^ were considered statistically significant (indicated in boldface). For detailed calculations of the traits, see Table S2. The numbers of samples for which subclass-specific glycosylation data were available can be found in Table S1.

### IgG Fc glycosylation associates with patient outcome in children below 4 years old.

**FIG 3:**
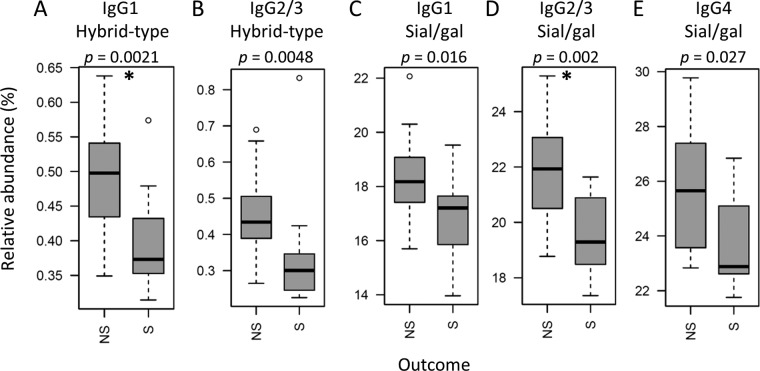
Levels of IgG Fc glycosylation features between meningococcal patients below the age of 4 years with severe (S) and nonsevere (NS) disease outcomes. Shown are box and whisker plots of the levels of (A) IgG1 hybrid-type glycans, (B) IgG2/3 hybrid-type glycans, (C) IgG1 sialylation per galactose, (D) IgG2/3 sialylation per galactose, and (E) IgG4 sialylation per galactose, where the boxes represent the interquartile range (IQR) and the whiskers 1.5× IQR. After multiple-testing correction, *P* values below 2.7 × 10^−3^ were considered statistically significant (indicated by an asterisk). The number of samples for which subclass-specific glycosylation data were available can be found in Table S1.

**FIG 4:**
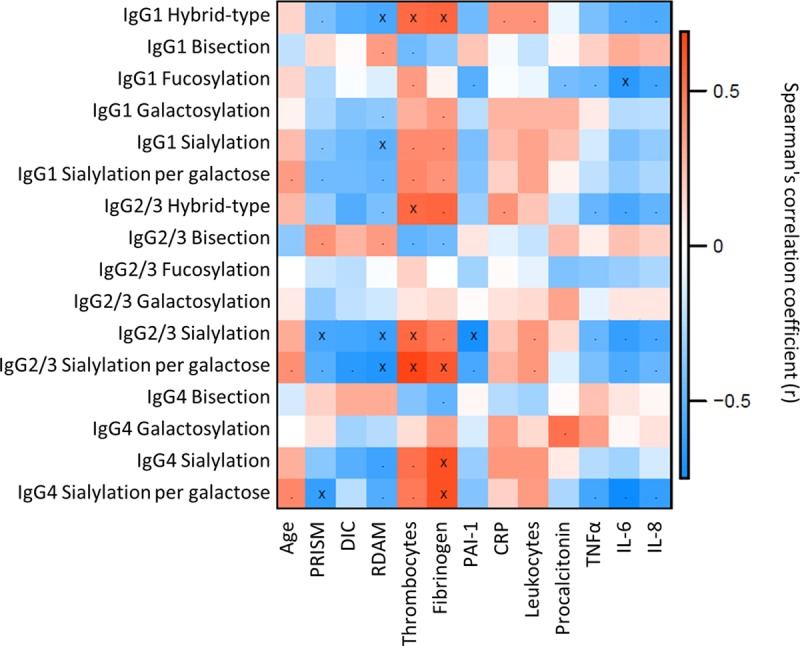
Correlation between IgG Fc glycosylation and clinical variables for meningococcal patients between 0 and 3.9 years old. The Spearman correlation coefficient is represented in red for a positive correlation and in blue for a negative correlation between the derived glycan trait and the outcome scores and inflammatory markers. Periods indicate *P* < 0.05, and crosses indicate *P* < 2.7 × 10^−3^ (α = 2.7 × 10^−3^, adjusted to allow an FDR of 5%).

IgG Fc glycosylation differences between cases and controls were most pronounced in the young children (below the age of 4 years), a group known to behave clinically differently from older meningococcus-infected patients (5, 33). The glycosylation differences between patients with severe disease outcome (death or amputation) and nonsevere disease outcome (full recovery) were only compared within this group (Table 3), as our cohort contained high data density in this age category (see Fig. S2 in the supplemental material). The abundance of hybrid-type structures on IgG1 was found to be overall low (below 1%); however, it appeared to be even lower in patients with a severe disease outcome (0.37%) compared to the patients that fully recovered (0.50%) (Fig. 3A). A similar observation was done for the hybrid-type structures on IgG2/3 (Fig. 3B), of which the levels correlated significantly with the levels of IgG1 hybrid-type glycans (see Fig. S3 in the supplemental material). In addition, IgG2/3 sialylation per galactose was lower in patients with a severe disease outcome (19.3%) compared to the patients with nonsevere outcome (21.9%) (Fig. 3D). A similar trend was also observed for sialylation per galactose on the other IgG subclasses (Fig. 3C and E), which correlated positively with the levels on IgG2/3 (Fig. S3). The IgG1 hybrid-type glycans and the sialylation per galactose on IgG2/3 associated negatively with the Rotterdam score (IgG1 hybrid-type correlation coefficient [*r*] = −0.6; IgG2/3 sialylation per galactose, *r* = −0.7 [Fig. 4; see Table S5 in the supplemental material]), known to be predictive for patient outcome (9). Also IgG1 and IgG2/3 sialylation associated negatively with the Rotterdam score (IgG1, *r* = −0.5; IgG2/3, *r* = −0.6). In addition, IgG2/3 sialylation and IgG4 sialylation per galactose associated negatively with the other clinically used predictive score, PRISM (*r* = −0.6 for both [Fig. 4]) (11), which correlated positively with the Rotterdam score (Fig. S3). The described associations were less pronounced in the total data set and were not present for the older pediatric patients (Fig. S3).

10.1128/mBio.00546-18.3 FIG S2  Glycosylation traits plotted versus age. Data points were plotted against age (years) per derived trait. Blue triangles, healthy controls; purple circles, meningococcal patients with nonsevere (NS) disease outcome; red crosses, meningococcal patients with severe (S) disease outcome. Download FIG S2, TIF file, 1.1 MB. Copyright © 2018 de Haan et al. 2018 de Haan et al. This content is distributed under the terms of the Creative Commons Attribution 4.0 International license.

10.1128/mBio.00546-18.4 FIG S3  Correlation heat maps. Correlation is shown between (A) IgG Fc glycosylation traits versus IgG Fc glycosylation traits for meningococcal sepsis patients between 0 and 4 years old, (B) clinical variables versus clinical variables for meningococcal sepsis patients between 0 and 4 years old, (C) IgG Fc glycosylation traits versus clinical variables for meningococcal patients between 0 and 18 years old, and (D) IgG Fc glycosylation traits versus clinical variables for meningococcal patients between 4 and 18 years old. The Spearman’s correlation coefficient is represented in red for a positive correlation and in blue for a negative correlation. Periods indicate *P* < 0.05, and crosses indicate *P* < 2.7 × 10^−3^ (α = 2.7 × 10^−3^ after 5% FDR correction). Download FIG S3, TIF file, 1.4 MB. Copyright © 2018 de Haan et al. 2018 de Haan et al. This content is distributed under the terms of the Creative Commons Attribution 4.0 International license.

10.1128/mBio.00546-18.10 TABLE S5  Correlation between IgG Fc glycosylation and clinical variables in the pediatric meningococcal patients. Spearman’s correlation tests were performed. After multiple-testing correction, *P* values below 2.7 × 10^−3^ were considered statistically significant (indicated in boldface). Download TABLE S5, PDF file, 0.4 MB. Copyright © 2018 de Haan et al. 2018 de Haan et al. This content is distributed under the terms of the Creative Commons Attribution 4.0 International license.

### Associations between IgG Fc glycosylation and inflammatory markers.

Various inflammatory markers were measured in the patient samples, including levels of thrombocytes, fibrinogen, PAI-1, CRP, leukocytes, procalcitonin, tumor necrosis factor alpha (TNF-α), and interleukin-6 (IL-6) and -8. Thrombocyte levels associated positively with IgG1 and IgG2/3 hybrid-type glycans in the young children (*r* = 0.6 for the hybrid-type glycans on both subclasses) (Fig. 4 and Table S5). The same effect was seen for IgG1 hybrid-type glycans and fibrinogen levels (*r* = 0.6). In addition, IgG2/3 sialylation per galactose correlated positively with fibrinogen and thrombocyte levels (*r* = 0.6 and 0.7, respectively). Furthermore, IgG2/3 sialylation associated negatively with PAI-1 levels (*r* = −0.7) and IgG1 fucosylation associated negatively with IL-6 (*r* = −0.7). In the older pediatric meningococcal patients, leukocyte levels were positively associated with IgG1 and IgG2/3 hybrid-type structures (*r* = 0.6 for both subclasses), and patients with lower CRP levels had lower levels of IgG2/3 hybrid-type structures (*r* = 0.7) (Fig. S3 and Table S5).

## DISCUSSION

### IgG Fc glycosylation in patients with meningococcal sepsis.

Pediatric meningococcal infections resulting in septic shock often occur in young and previously healthy children (1). The reason why young children are more susceptible to this severe infection and the factors that determine the outcome of disease are largely unknown (5). In this study, we analyzed the IgG Fc glycosylation of children admitted to the PICU with meningococcal sepsis. Changes in IgG Fc glycosylation are known to have a large influence on the effector function of the antibody and are associated with various inflammatory conditions (18, 22–24, 28).

We found IgG1 Fc fucosylation to be lower in patients than in age-matched healthy controls. As previous research showed no sex-related IgG glycosylation effects in healthy children between 3 months and 17 years (31), the boys and girls in the present study were not assessed separately. The observed difference between cases and controls was more pronounced in children below the age of 4 years. In addition, in children of this age category, IgG1 Fc bisection was higher in patients than in controls. Previous studies reported a different disease course and mortality rate in very young children, showing the relevance of studying this patient group separately from older pediatric meningococcal sepsis patients (1, 5). Additionally, we found that the cytokine levels in our cohort tended to be higher in younger patients than that in the older ones. This might be explained by a trend of higher illness severity in young patients and consequently higher cytokine levels. During the maturation of the immune system, when children start to produce their own IgG molecules, the Fc fucosylation is relatively high compared to that in older children (above the age of 4 years), while the Fc bisection is relatively low (31). In young children with meningococcal sepsis, a change in the glycosylation of a disease-specific subset of their IgGs might be induced by the meningococcal infection itself in an early stage of the disease. IgG glycosylation is able to change quickly, as shown in patients experiencing acute systemic inflammation after cardiac surgery, where part of the patients showed an increased antibody galactosylation the first day after surgery (34). In addition, studies in mice showed that Fc sialylation could be regulated dynamically, by the interplay of soluble sialyltransferases and the accumulation of platelets providing CMP-sialic acid (35, 36). The fact that sialyl- and galactosyltransferases are also circulating in the human plasma suggests that glycosylation might be dynamically regulated in humans too (37).

Alternatively, low Fc fucosylation and high Fc bisection in young patients rather reflect the extent of exposure to previous infections and subsequent adaptive immune responses. Antigen-specific IgG Fc glycosylation was previously shown to differ substantially from the glycosylation of the total pool of IgG. For example, gp120-specific IgG in HIV-infected patients displays significantly lower Fc fucosylation than the total pool of IgG in the infected patients (25). Furthermore, in alloimmune cytopenias also low levels of Fc fucosylation were observed in anti-HPA-1a and anti-RhD IgGs, compared to the total IgG pool (26, 27). Low IgG1 Fc fucosylation (i.e., high afucosylation) enhances binding of IgG to FcγRIIIa and FcγRIIIb 20- to 100-fold (22, 38), thereby increasing ADCC (18, 22). The 2-fold higher abundance of afucosylated IgGs that we observed for meningococcal sepsis patients might reflect the upregulation of antigen-specific groups of IgG. For IgG specific for meningococcal outer membrane vesicle (OMV) antigens obtained after OMV vaccination, no change in fucosylation was seen over time (39). However, no comparison was made between total IgG before and after vaccination, and glycosylation changes on antigen-specific IgG might be substantially different between vaccinated and naturally infected individuals. We speculate that the low IgG fucosylation observed in young meningococcal patients may reflect a history of exposure to (viral) infections, or in a broader sense antigenic stimuli, resulting in the buildup of low-fucosylation IgG against the respective antigens, which manifests itself in a shift of the total IgG pool toward lower fucosylation. Hence, the low fucosylation may be a marker of a history of infections which may, e.g., reflect a certain proneness to viral or other infectious diseases in these children.

Future studies should elucidate whether skewed IgG Fc glycosylation featuring low fucosylation and high bisection can be identified on meningococcus-specific IgG and whether the deviations from normal are induced by the meningococcal infection or were already present in the children before infection.

### IgG Fc glycosylation associates with illness severity.

Young patients with severe disease, defined by death or need for amputation, have a lower level of IgG1 hybrid-type glycans and IgG2/3 sialylation per galactose when admitted to the PICU. In addition, these glycosylation features correlate negatively with illness severity, as measured by the Rotterdam score, as well as positively with previously determined predictive factors in meningococcal sepsis, namely, levels of thrombocytes and fibrinogen (9), which correlate negatively with severity. Thus, our data suggest that IgG1 hybrid-type glycans and IgG2/3 sialylation per galactose could be a predictor for meningococcal sepsis severity.

Hybrid-type glycans are known to be present on human IgG-Fc in minor amounts, and not much is known about their function (40, 41). We show here, for the first time, that the level of hybrid-type glycans on both IgG1 and IgG2/3 correlates negatively with age in healthy children. This is likely connected to maturation of the immune system as hybrid-type glycans are precursors of the usually found complex-type glycans on IgG and might originate from immature B cells.

In the young patients, IgG2/3 Fc sialylation seems to change independently from the level of Fc galactosylation (serving as a substrate for sialylation), which is likely an effect of the higher availability of sialyltransferase ST6Gal1 or the increased presence of the substrate CMP-sialic acid, either inside the Golgi apparatus or outside the cell (35, 36, 42). IgG1 Fc sialylation is known to modulate antibody binding to C1q, and subsequent CDC, either positively (22) or negatively (23). This discrepancy is suggested to be caused by the spatial distribution of the monoclonal antibodies on the cell surface, which depends on the monoclonal antibody studied.

Similar to the glycosylation differences observed between cases and controls, the question is whether the low levels of hybrid-type glycans and sialylation per galactose are emerging during the course of the disease (and are thus meningococcal sepsis specific) or were already present on (a fraction of) the IgG Fc of patients appearing to have a severe disease outcome. In both situations, the alterations can either be the cause of the severe outcome or a bystander effect. In either situation, IgG Fc glycosylation features have the potential to be used to predict meningococcal sepsis outcome in very young patients, which should be validated in larger study populations.

Interestingly, IgG1 fucosylation associated negatively with IL-6 levels in patients below the age of 4 years, while previous studies in healthy adults showed a positive correlation between IL-6 levels and fucosylation (43). IL-6 levels have been shown before to be elevated with meningococcal sepsis and to have a potential role in outcome prediction (44). Furthermore, none of the glycosylation features are associated with levels of CRP in the young patients, while CRP in the healthy adult population does associate with IgG Fc galactosylation (negatively) and Fc fucosylation (positively) (43). Low CRP levels at the time of admission at the PICU are a known predictor of mortality rate in meningococcal sepsis (9), indicating that the prediction based on CRP is grounded on different mechanisms than the prediction based on glycosylation features. This opens possibilities to combine these factors for an improved prediction tool.

### Conclusion.

We found IgG1 fucosylation and bisection to be associated with meningococcal sepsis in children below the age of 4 years. Within these young patients, we found IgG1 hybrid-type glycans and IgG2/3 sialylation per galactose correlated with the severity of the disease. Further research is needed to determine whether the observed glycosylation differences between patients and controls are a result of the meningococcal infection itself or rather associated with increased susceptibility to meningococcal septic shock. Furthermore, glycosylation changes associated with illness severity have the potential to be used as outcome predictors, which should be validated in larger study populations.

## MATERIALS AND METHODS

### Patients and controls.

In the current retrospective study, plasma or serum samples of prospective cohorts of children with meningococcal sepsis were used. Samples were collected from patients recruited for pediatric meningococcal sepsis studies (1988 to 2005) at the PICU of Erasmus MC-Sophia Children’s Hospital (Rotterdam, The Netherlands) (9, 45–47). These studies were conducted in accordance with the Declaration of Helsinki and Good Clinical Practice guidelines. All individual meningococcal studies as well as the present study were approved by the Ethical Committee of Erasmus MC (MEC-2015-497), and written informed consent was obtained from parents or legal guardians.

Blood samples from 60 children with meningococcal sepsis were taken within 6 h after admission to the PICU. All patients fulfilled internationally agreed criteria for sepsis (48). Most patients already received antibiotic treatment at the moment of sampling and had a central venous catheter *in situ*. In addition, treatment and medication assisting in resuscitation were generally given (such as fluids and inotropes). Samples were processed on ice and were stored at −80°C until analysis. The sample types comprised a variety of serum, citrate plasma, heparin plasma, and EDTA plasma. For several patients, multiple materials taken at the same time point were available.

Plasma samples of 46 healthy controls, selected to be in the same age range as the patients, were collected in accordance with the Declaration of Helsinki and Good Clinical Practice guidelines (31, 49). The collection of the samples was approved by the Ethical Committee of Erasmus MC (MEC-2005-137), and written informed consent was obtained from parents or legal guardians.

### Clinical data collection.

Various clinical data were collected, illness severity was indicated by Pediatric Risk of Mortality (PRISM) (11), predicted death based on the Rotterdam score (9), and the disseminated intravascular coagulation (DIC) score (50). Coagulation (thrombocytes, fibrinogen, and PAI-1) and inflammation (leukocytes, procalcitonin, CRP, tumor necrosis factor alpha [TNF-α], interleukins [IL-6 and -8]) markers were measured for clinical reasons or were obtained in previous meningococcal sepsis studies (9, 45, 47). Patients were classified to have died if death occurred during the PICU stay. The need for amputation and/or the occurrence of death during the PICU stay were together classified as a severe disease outcome.

### Chemicals.

Disodium hydrogen phosphate dihydrate (Na_2_HPO_4_⋅2H_2_O), potassium dihydrogen phosphate (KH_2_PO_4_), NaCl, and trifluoroacetic acid were purchased from Merck (Darmstadt, Germany). Formic acid, ammonium bicarbonate, and TPCK (tosylsulfonyl phenylalanyl chloromethyl ketone)-treated trypsin from bovine pancreas were obtained from Sigma-Aldrich (Steinheim, Germany). Furthermore, high-performance liquid chromatography (HPLC) SupraGradient acetonitrile (ACN) was obtained from Biosolve (Valkenswaard, The Netherlands), and ultrapure deionized water (MQ) was generated by the Purelab Ultra, maintained at 18.2 MΩ (Veolia Water Technologies Netherlands BV, Ede, The Netherlands). Phosphate-buffered saline (PBS [pH 7.3]) was made in house, containing 5.7 g/liter Na_2_HPO_4_⋅2H_2_O, 0.5 g/liter KH_2_PO_4_, and 8.5 g/liter NaCl.

### IgG isolation and glycopeptide preparation.

The 98 clinical samples and 46 healthy control samples were randomized in a 96-well plate format, together with 32 VisuCon pooled plasma standards (Affinity BioLogicals, Inc., Ancaster, Ontario, Canada [8 per plate]) and 16 PBS blanks (minimally 2 per plate). Randomization was performed in a supervised way, selecting an optimal distribution of age, sex, and case/control ratio per plate. IgG was isolated using protein G affinity beads (GE Healthcare, Uppsala, Sweden) as described previously (51). Briefly, 2 µl of plasma was incubated with 15 µl of beads in 100 µl of PBS for 1 h with agitation. Beads were then washed three times with 200 µl of PBS and three times with 200 µl of MQ, after which the antibodies were eluted with 100 µl 100 mM formic acid. Eluates were dried for 2 h at 60°C in a vacuum concentrator and dissolved in 40 µl 25 mM ammonium bicarbonate containing 25 ng/µl trypsin. Samples were shaken for 10 min and incubated at 37°C for 17 h.

### LC-MS analysis of glycopeptides.

The IgG digest was separated and analyzed by an Ultimate 3000 high-performance liquid chromatography (HPLC) system (Dionex Corporation, Sunnyvale, CA) coupled to a Maxis Impact HD quadrupole time of flight mass spectrometry (QTOF-MS) device (Bruker Daltonics) as described before (51) and explained in detail in Text S1 in the supplemental material.

10.1128/mBio.00546-18.1 TEXT S1  Supplemental materials and methods. Download TEXT S1, PDF file, 0.3 MB. Copyright © 2018 de Haan et al. 2018 de Haan et al. This content is distributed under the terms of the Creative Commons Attribution 4.0 International license.

### Data processing.

The raw liquid chromatography-mass spectrometry (LC-MS) data were extracted and curated using LacyTools v0.0.7.2 as described previously (51, 52); cohort-specific parameters are provided in the supplemental materials and methods (Text S1). Using the described separation methods, glycopeptides with the same peptide portion coeluted. This resulted in three glycopeptide clusters: one for IgG1, one for IgG4, and one for the combination of IgG2 and IgG3. As the study population was mainly of Caucasian ancestry, the tryptic Fc glycopeptides of IgG2 and -3 were assumed to have identical masses and could therefore not be distinguished by our profiling method. However, it is possible that for part of the samples, the IgG3 glycopeptides are coanalyzed with the ones of IgG4, due to the presence of different IgG3 allotypes (53). All chromatograms were aligned based on the exact mass and the average retention time of the three most abundant glycoforms of each IgG subclass. After alignment, sum spectra were created per glycopeptide cluster and then calibrated based on at least five glycopeptides per cluster with a signal/noise (S/N) ratio higher than 9. For the targeted extraction, analyte lists were created by manual annotation of summed spectra per biological class (healthy or meningococcal sepsis), covering both doubly charged and triply charged species. Compositional assignments were made on the basis of accurate mass and literature (20, 54). Glycopeptide signals were integrated by including enough isotopomers to cover at least 95% of the area of the isotopic envelope. Spectra were excluded from further analysis when the total spectrum intensity was below 10 times the average spectrum intensity of the blanks. In this way, no spectra were excluded for IgG1, 15 spectra were excluded for IgG2/3, and 29 spectra were excluded for IgG4. Analytes were included in the final data analysis when their average S/N ratio (calculated per biological class) was above 9, their isotopic pattern did not, on average, deviate more than 20% from the theoretical pattern, and their average mass error was within ±10 ppm. This resulted in the extraction of 22 IgG1, 15 IgG2/3, and 10 IgG4 glycoforms (Table S2).

### Data analysis.

The absolute intensities of the extracted glycoforms were total area normalized per IgG subclass, and derived glycan traits were calculated based on specific glycosylation features (Table 2 and Table S2). Data quality was evaluated based on the 32 pooled plasma standards that were randomly included in the cohort. These resulted in highly repeatable profiles showing median relative standard deviations of 3.6% for the IgG1 glycopeptides, 2.9% for the IgG2/3 glycopeptides, and 2.4% for the IgG4 glycopeptides (see Fig. S4 in the supplemental material). For 10 patients, serum, citrate plasma, and EDTA plasma of the same time point were available. For these samples, the relative intensities of the individual glycoforms were averaged over the different materials and relative standard deviations were calculated and compared to the standard deviations obtained from the technical replicates. This revealed in general no higher relative standard deviation over the different materials than over the technical replicates (Fig. S4). For patients who had different materials available at the same time point, the data of the samples were averaged. Statistical analysis was performed using R 3.1.2 (R Foundation for Statistical Computing, Vienna, Austria) and RStudio 0.98.1091 (RStudio, Inc.). First, outliers were removed, which were defined as values outside the 99% confidence interval per biological group (healthy or meningococcal sepsis). Samples were excluded from further statistical analysis when two or more of the derived traits per IgG subclass were marked as outliers. This resulted in the exclusion of one IgG1 sample, one IgG2/3 sample, and two IgG4 samples. The derived glycosylation traits per subclass were tested to correlate with age and the continuous clinical variables using Spearman’s correlation test. Mann-Whitney *U* tests were performed to assess glycosylation differences between cases and controls and between patients with severe and nonsevere disease outcomes. All statistical tests were performed both on the whole data set and on the subsets of children below the age of 4 years and above the age of 4. The tests for disease outcome were exclusively performed on the younger age category (below 4 years). The significance threshold (α) was adjusted for multiple testing by the Benjamini Hochberg false-discovery rate (FDR) method, with an FDR of 5%. This resulted in α = 0.0027 throughout the study.

10.1128/mBio.00546-18.5 FIG S4  Method robustness. Relative abundances of the extracted IgG1 (A), IgG2/3 (B), and IgG4 (C) glycoforms in the 32 pooled plasma standards included in the cohort (final bar, labeled “Standard”) and for 10 patients for which three materials (serum, citrate plasma, and EDTA plasma) collected at the same time point were available (first 10 bars, labeled “P1” to “P10”). Shown are the average relative abundances and standard deviations over all technical replicates (Standard) or over the measurements from the three materials (P1 to P10). The standards reveal highly repeatable profiles throughout the cohort measurements. The profiling in different materials shows relative standard deviations over the three materials in general not to be larger than the relative standard deviations over the technical replicates. H, hexose; N, *N*-acetylhexosamine; F, fucose; S, *N*-acetylneuraminic acid. Download FIG S4, TIF file, 0.4 MB. Copyright © 2018 de Haan et al. 2018 de Haan et al. This content is distributed under the terms of the Creative Commons Attribution 4.0 International license.
