# Supplementary figures and images for: Differences in IgG Fc Glycosylation Are Associated with Outcome of Pediatric Meningococcal Sepsis
Source: mBio. 2018 Jun 19;9(3):e00546-18. doi: 10.1128/mBio.00546-18 (PMC6016251; doi:10.1128/mBio.00546-18)

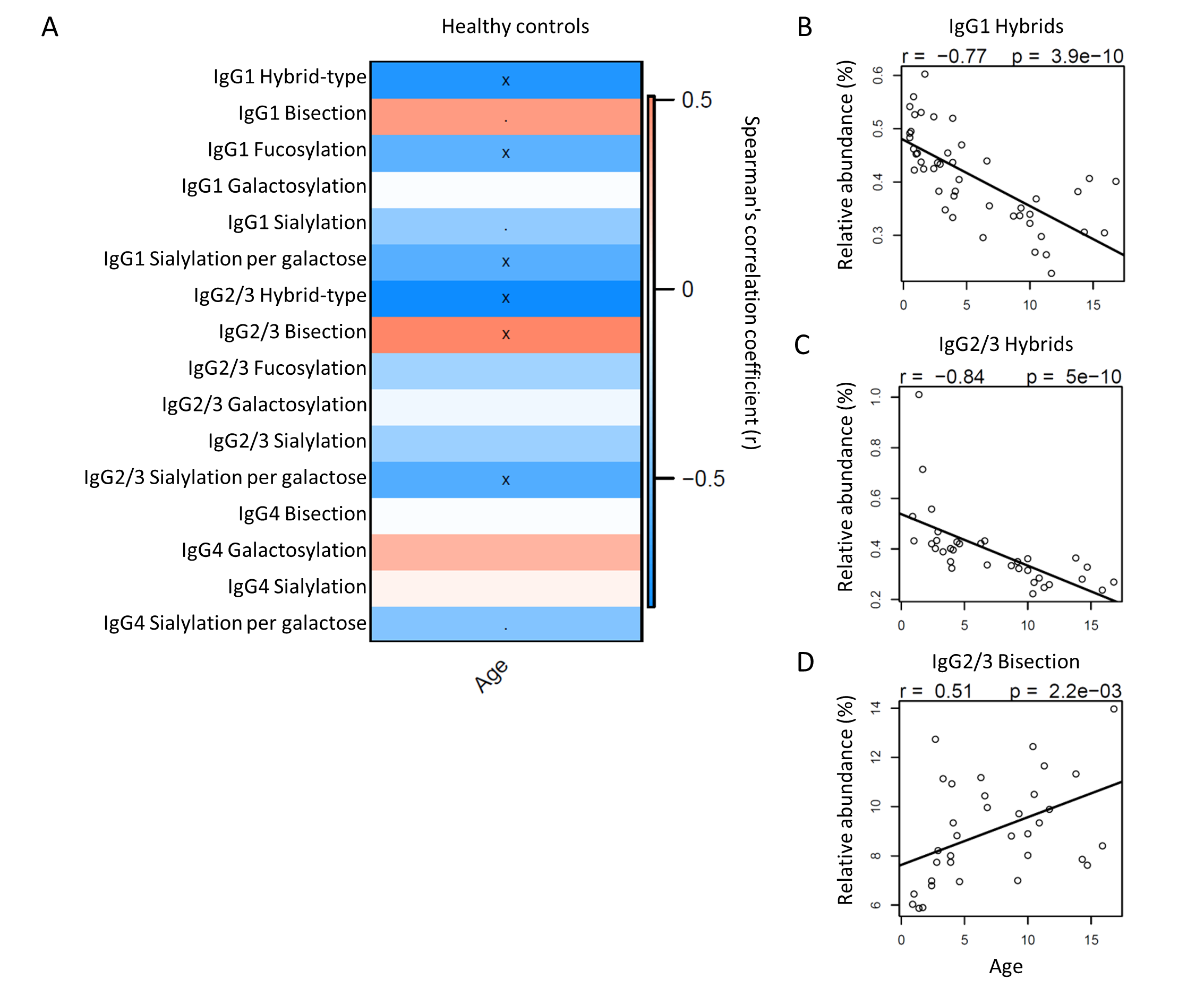

Supplement: FIG S1 [file mbo003183922sf1.tif]

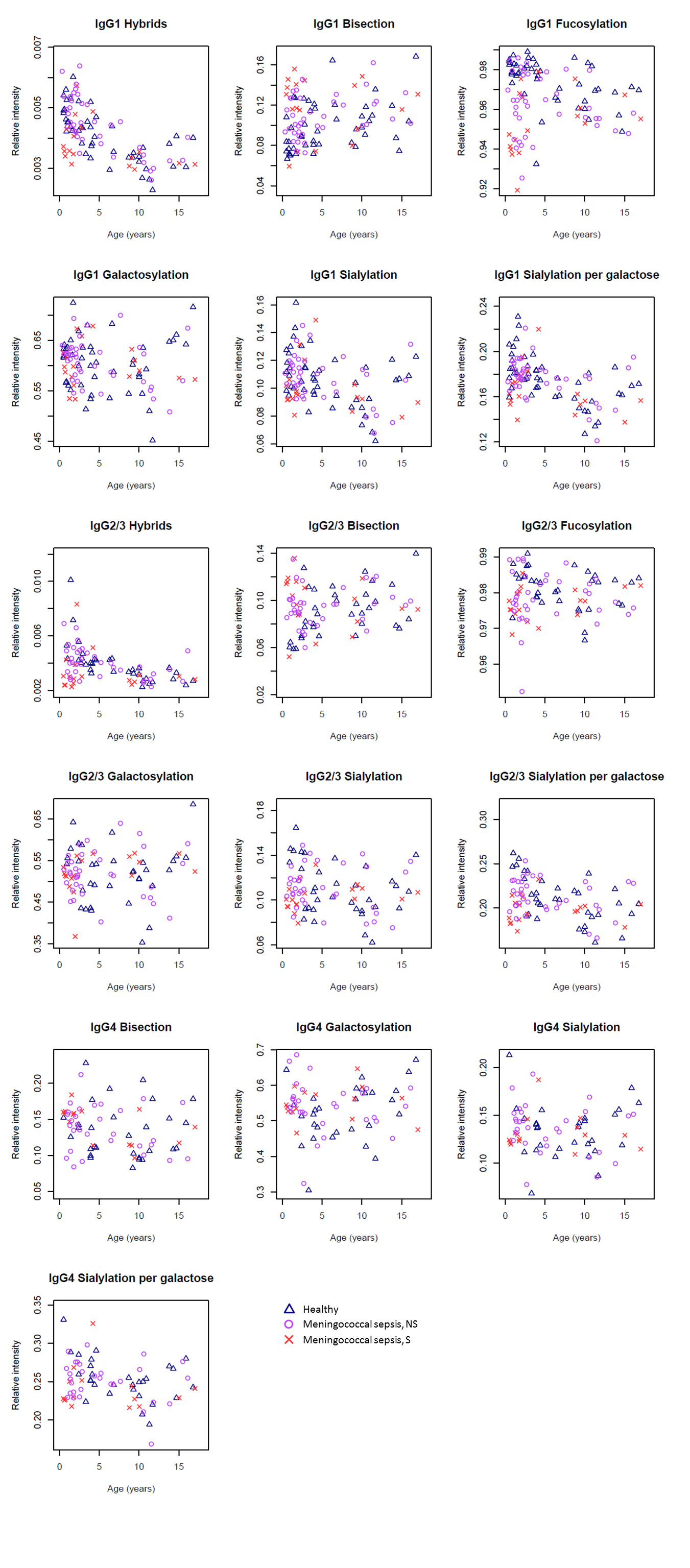

Supplement: FIG S2 [file mbo003183922sf2.tif]

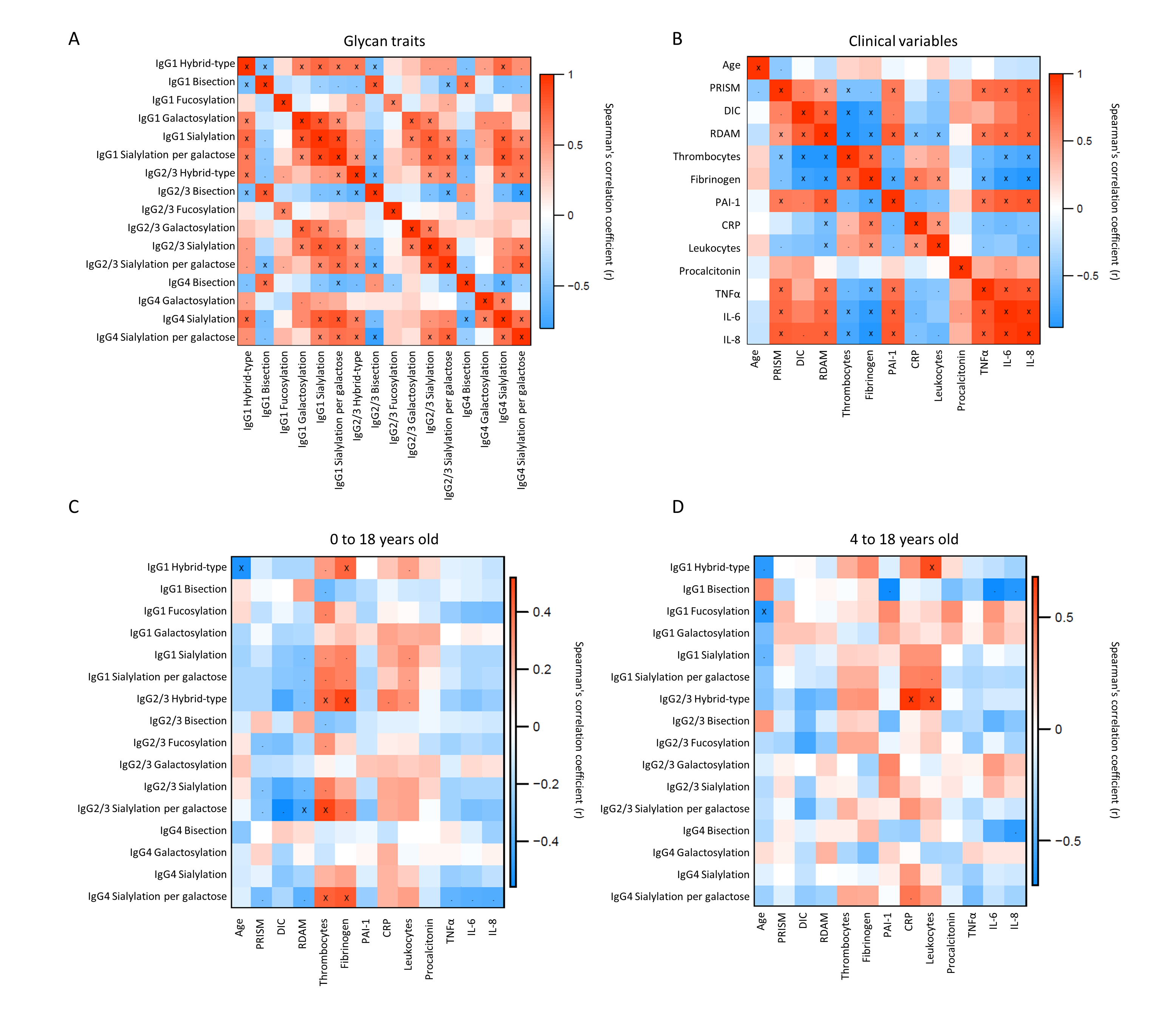

Supplement: FIG S3 [file mbo003183922sf3.tif]

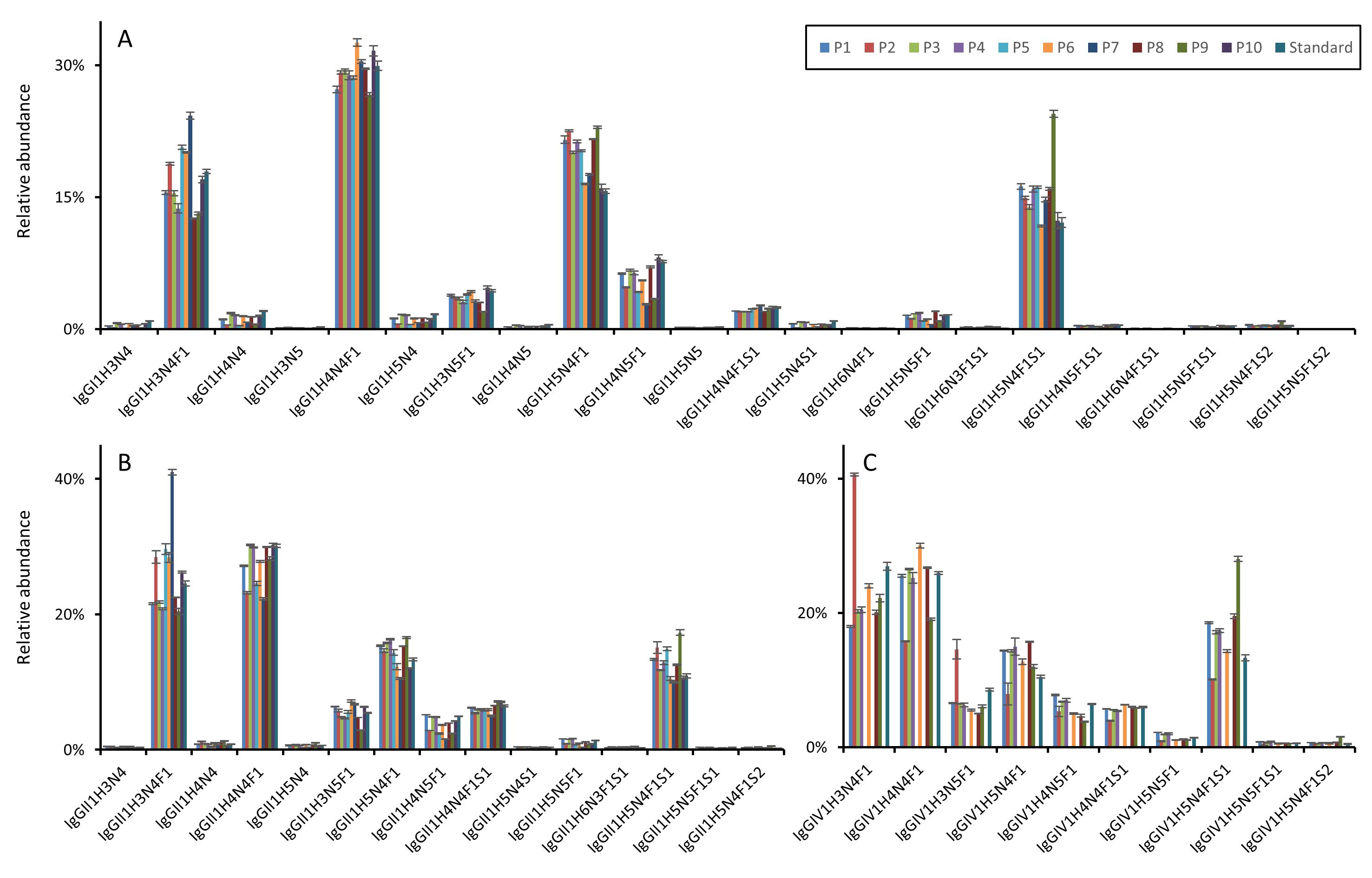

Supplement: FIG S4 [file mbo003183922sf4.tif]
