# Supplementary material for: Differences in IgG Fc Glycosylation Are Associated with Outcome of Pediatric Meningococcal Sepsis
Source: mBio. 2018 Jun 19;9(3):e00546-18. doi: 10.1128/mBio.00546-18 (PMC6016251; doi:10.1128/mBio.00546-18)
Supplement: TABLE S1 [file mbo003183922st1.pdf]

Clinical data	Number of patients for which specific clinical data is present		
	All patients	Patients <4 years	Patients ≥4 years
Total number of samples	60	37	22
Age	59	37	22
Sex	59	37	22
Illness severity			
PRISM score	58	36	22
P (death Rotterdam)	54	34	20
DIC score	22	14	8
Coagulation markers			
Thrombocytes	58	36	22
Fibrinogen	52	31	21
PAI-1	35	20	15
Inflammatory markers			
Leukocytes	58	36	22
C-reactive protein	56	36	22
Procalcitonin	38	24	14
TNFα	40	23	17
Interleukin-6	35	20	15
Interleukin-8	35	20	15
Outcome			
Mortality	59	37	22
Amputation	59	37	22
Severe outcome	59	37	22

Sample group	Number of samples for which subclass-specific glycosylation data is present		
	IgG1	IgG2/3	IgG4
Healthy	46	34	29
Healthy <4 years	24	12	8
Healthy ≥4 years	22	22	21
Patients	60	57	48
Patients <4 years	37	34	26
Patients ≥4 years	22	22	21
Patients severe outcome	19	19	16
Patients severe outcome <4 years	12	12	9
Patients severe outcome ≥4 years	7	7	7
Patients non-severe outcome	40	37	31
Patients non-severe outcome <4 years	25	22	17
Patients non-severe outcome ≥4 years	15	15	14
