# Supplementary material for: Differences in IgG Fc Glycosylation Are Associated with Outcome of Pediatric Meningococcal Sepsis
Source: mBio. 2018 Jun 19;9(3):e00546-18. doi: 10.1128/mBio.00546-18 (PMC6016251; doi:10.1128/mBio.00546-18)
Supplement: TABLE S2 [file mbo003183922st2.pdf]

Composition	Depiction	[M+3H]$^{3+}$ when extracted		
		IgG1	IgG2/3	IgG4
**H3N4**	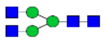	829.997	819.334	
**H3N4F1**	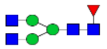	878.683	868.02	873.351
**H4N4**	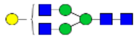	884.015	873.351	
**H3N5**	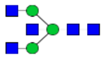	897.694		
**H4N4F1**	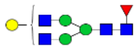	932.7	922.037	927.369
**H5N4**	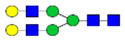	938.032	927.369	
**H3N5F1**	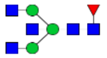	946.376	935.713	941.044
**H4N5**	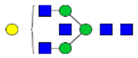	951.708		
**H5N4F1**	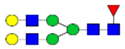	986.718	976.055	981.386
**H4N5F1**	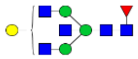	1000.394	989.73	995.062
**H5N5**	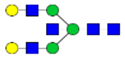	1005.729		
**H4N4F1S1**	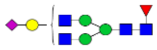	1029.732	1019.069	1024.401
**H5N4S1**	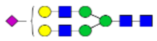	1035.064	1024.401	
**H6N4F1**	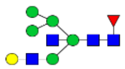	1040.74		
**H5N5F1**	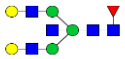	1054.411	1043.748	1049.08
**H6N3F1S1**	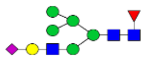	1070.074	1059.411	
**H5N4F1S1**	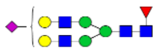	1083.75	1073.087	1078.418
**H4N5F1S1**	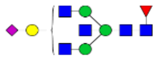	1097.425		

**H6N4F1S1**	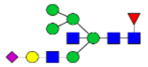	1137.771		
**H5N5F1S1**	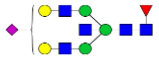	1151.443	1140.78	1146.111
**H5N4F1S2**	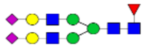	1180.782	1170.118	1175.45
**H5N5F1S2**	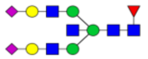	1248.479		

Derived trait	Depiction	Description	Calculation
**IgG1 Hybrid-type**	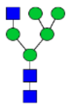	Fraction of hybrid glycans on IgG1	(IgG1 H6N4F1 + IgG1 H6N3F1S1 + IgG1 H6N4F1S1) / (IgG1 H3N4 + IgG1 H3N4F1 + IgG1 H4N4 + IgG1 H3N5 + IgG1 H4N4F1 + IgG1 H5N4 + IgG1 H3N5F1 + IgG1 H4N5 + IgG1 H5N4F1 + IgG1 H4N5F1 + IgG1 H5N5 + IgG1 H4N4F1S1 + IgG1 H5N4S1 + IgG1 H6N4F1 + IgG1 H5N5F1 + IgG1 H6N3F1S1 + IgG1 H5N4F1S1 + IgG1 H4N5F1S1 + IgG1 H6N4F1S1 + IgG1 H5N5F1S1 + IgG1 H5N4F1S2 + IgG1 H5N5F1S2)
**IgG1 Bisection**	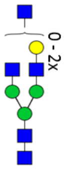	Bisection on IgG1	(IgG1 H3N5 + IgG1 H3N5F1 + IgG1 H4N5 + IgG1 H4N5F1 + IgG1 H5N5 + IgG1 H5N5F1 + IgG1 H4N5F1S1 + IgG1 H5N5F1S1 + IgG1 H5N5F1S2 + IgG1 H6N4F1 + IgG1 H6N4F1S1) / (IgG1 H3N4 + IgG1 H3N4F1 + IgG1 H4N4 + IgG1 H3N5 + IgG1 H4N4F1 + IgG1 H5N4 + IgG1 H3N5F1 + IgG1 H4N5 + IgG1 H5N4F1 + IgG1 H4N5F1 + IgG1 H5N5 + IgG1 H4N4F1S1 + IgG1 H5N4S1 + IgG1 H6N4F1 + IgG1 H5N5F1 + IgG1 H6N3F1S1 + IgG1 H5N4F1S1 + IgG1 H4N5F1S1 + IgG1 H6N4F1S1 + IgG1 H5N5F1S1 + IgG1 H5N4F1S2 + IgG1 H5N5F1S2)
**IgG1 Fucosylation**	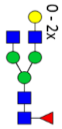	Fucosylation on IgG1	(IgG1 H3N4F1 + IgG1 H4N4F1 + IgG1 H3N5F1 + IgG1 H5N4F1 + IgG1 H4N5F1 + IgG1 H4N4F1S1 + IgG1 H6N4F1 + IgG1 H5N5F1 + IgG1 H6N3F1S1 + IgG1 H5N4F1S1 + IgG1 H4N5F1S1 + IgG1 H6N4F1S1 + IgG1 H5N5F1S1 + IgG1 H5N4F1S2 + IgG1 H5N5F1S2) / (IgG1 H3N4 + IgG1 H3N4F1 + IgG1 H4N4 + IgG1 H3N5 + IgG1 H4N4F1 + IgG1 H5N4 + IgG1 H3N5F1 + IgG1 H4N5 + IgG1 H5N4F1 + IgG1 H4N5F1 + IgG1 H5N5 + IgG1 H4N4F1S1 + IgG1 H5N4S1 + IgG1 H6N4F1 + IgG1 H5N5F1 + IgG1 H6N3F1S1 + IgG1 H5N4F1S1 + IgG1 H4N5F1S1 + IgG1 H6N4F1S1 + IgG1 H5N5F1S1 + IgG1 H5N4F1S2 + IgG1 H5N5F1S2)
**IgG1 Galactosylation**	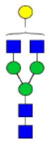	Galactosylation per antenna of diantennary glycans on IgG1	(1/2 * (IgG1 H4N4 + IgG1 H4N4F1 + IgG1 H4N5 + IgG1 H4N5F1 + IgG1 H4N4F1S1 + IgG1 H4N5F1S1) + (IgG1 H5N4 + IgG1 H5N4F1 + IgG1 H5N5 + IgG1 H5N4S1 + IgG1 H5N5F1 + IgG1 H5N4F1S1 + IgG1 H5N5F1S1 + IgG1 H5N4F1S2 + IgG1 H5N5F1S2)) / (IgG1 H3N4 + IgG1 H3N4F1 + IgG1 H4N4 + IgG1 H3N5 + IgG1 H4N4F1 + IgG1 H5N4 + IgG1 H3N5F1 + IgG1 H4N5 + IgG1 H5N4F1 + IgG1 H4N5F1 + IgG1 H5N5 + IgG1 H4N4F1S1 + IgG1 H5N4S1 + IgG1 H5N5F1 + IgG1 H5N4F1S1 + IgG1 H4N5F1S1 + IgG1 H5N5F1S1 + IgG1 H5N4F1S2 + IgG1 H5N5F1S2)
**IgG1 Sialylation**	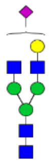	Sialylation per antenna of diantennary glycans on IgG1	(1/2 * (IgG1 H4N4F1S1 + IgG1 H5N4S1 + IgG1 H5N4F1S1 + IgG1 H4N5F1S1 + IgG1 H5N5F1S1) + (IgG1 H5N4F1S2 + IgG1 H5N5F1S2)) / (IgG1 H3N4 + IgG1 H3N4F1 + IgG1 H4N4 + IgG1 H3N5 + IgG1 H4N4F1 + IgG1 H5N4 + IgG1 H3N5F1 + IgG1 H4N5 + IgG1 H5N4F1 + IgG1 H4N5F1 + IgG1 H5N5 + IgG1 H4N4F1S1 + IgG1 H5N4S1 + IgG1 H5N5F1 + IgG1 H5N4F1S1 + IgG1 H4N5F1S1 + IgG1 H5N5F1S1 + IgG1 H5N4F1S2 + IgG1 H5N5F1S2)
**IgG1 Sialylation per galactose**	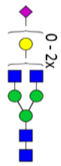	Sialylation per galactose of diantennary glycans on IgG1	IgG1 Sialylation / IgG1 Galactosylation
**IgG2/3 Hybrid-type**	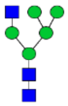	Fraction of hybrid glycans on IgG2/3	(IgG2/3 H6N3F1S1) / (IgG2/3 H3N4 + IgG2/3 H3N4F1 + IgG2/3 H4N4 + IgG2/3 H4N4F1 + IgG2/3 H5N4 + IgG2/3 H3N5F1 + IgG2/3 H5N4F1 + IgG2/3 H4N5F1 + IgG2/3 H4N4F1S1 + IgG2/3 H5N4S1 + IgG2/3 H5N5F1 + IgG2/3 H6N3F1S1 + IgG2/3 H5N4F1S1 + IgG2/3 H5N5F1S1 + IgG2/3 H5N4F1S2)

**IgG2/3 Bisection**	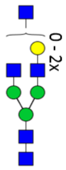	Bisection on IgG2/3	(IgG2/3 H3N5F1 + IgG2/3 H4N5F1 + IgG2/3 H5N5F1 + IgG2/3 H5N5F1S1) / (IgG2/3 H3N4 + IgG2/3 H3N4F1 + IgG2/3 H4N4 + IgG2/3 H4N4F1 + IgG2/3 H5N4 + IgG2/3 H3N5F1 + IgG2/3 H5N4F1 + IgG2/3 H4N5F1 + IgG2/3 H4N4F1S1 + IgG2/3 H5N4S1 + IgG2/3 H5N5F1 + IgG2/3 H6N3F1S1 + IgG2/3 H5N4F1S1 + IgG2/3 H5N5F1S1 + IgG2/3 H5N4F1S2)
**IgG2/3 Fucosylation**	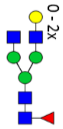	Fucosylation on IgG2/3	(IgG2/3 H3N4F1 + IgG2/3 H4N4F1 + IgG2/3 H3N5F1 + IgG2/3 H5N4F1 + IgG2/3 H4N5F1 + IgG2/3 H4N4F1S1+ IgG2/3 H5N5F1 + IgG2/3 H6N3F1S1 + IgG2/3 H5N4F1S1 + IgG2/3 H5N5F1S1 + IgG2/3 H5N4F1S2) / (IgG2/3 H3N4 + IgG2/3 H3N4F1 + IgG2/3 H4N4 + IgG2/3 H4N4F1 + IgG2/3 H5N4 + IgG2/3 H3N5F1 + IgG2/3 H5N4F1 + IgG2/3 H4N5F1 + IgG2/3 H4N4F1S1 + IgG2/3 H5N4S1 + IgG2/3 H5N5F1 + IgG2/3 H6N3F1S1 + IgG2/3 H5N4F1S1 + IgG2/3 H5N5F1S1 + IgG2/3 H5N4F1S2)
**IgG2/3 Galactosylation**	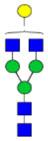	Galactosylation per antenna of diantennary glycans on IgG2/3	(1/2 * (IgG2/3 H4N4 + IgG2/3 H4N4F1 + IgG2/3 H4N5F1 + IgG2/3 H4N4F1S1) + (IgG2/3 H5N4 + IgG2/3 H5N4F1 + IgG2/3 H5N4S1 + IgG2/3 H5N5F1 + IgG2/3 H5N4F1S1 + IgG2/3 H5N5F1S1 + IgG2/3 H5N4F1S2)) / (IgG2/3 H3N4 + IgG2/3 H3N4F1 + IgG2/3 H4N4 + IgG2/3 H4N4F1 + IgG2/3 H5N4 + IgG2/3 H3N5F1 + IgG2/3 H5N4F1 + IgG2/3 H4N5F1 + IgG2/3 H4N4F1S1 + IgG2/3 H5N4S1 + IgG2/3 H5N5F1 + IgG2/3 H5N4F1S1 + IgG2/3 H5N5F1S1 + IgG2/3 H5N4F1S2)
**IgG2/3 Sialylation**	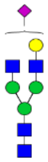	Sialylation per antenna of diantennary glycans on IgG2/3	(1/2 * (IgG2/3 H4N4F1S1 + IgG2/3 H5N4S1 + IgG2/3 H5N4F1S1 + IgG2/3 H5N5F1S1) + (IgG2/3 H5N4F1S2)) / (IgG2/3 H3N4 + IgG2/3 H3N4F1 + IgG2/3 H4N4 + IgG2/3 H4N4F1 + IgG2/3 H5N4 + IgG2/3 H3N5F1 + IgG2/3 H5N4F1 + IgG2/3 H4N5F1 + IgG2/3 H4N4F1S1 + IgG2/3 H5N4S1 + IgG2/3 H5N5F1 + IgG2/3 H5N4F1S1 + IgG2/3 H5N5F1S1 + IgG2/3 H5N4F1S2)
**IgG2/3 Sialylation per galactose**	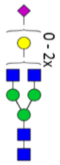	Sialylation per galactose of diantennary glycans on IgG2/3	IgG2/3 Sialylation / IgG2/3 Galactosylation
**IgG4 Bisection**	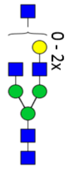	Bisection on IgG4	(IgG4 H3N5F1 + IgG4 H4N5F1 + IgG4 H5N5F1 + IgG4 H5N5F1S1) / (IgG4 H3N4F1 + IgG4 H4N4F1 + IgG4 H3N5F1 + IgG4 H5N4F1 + IgG4 H4N5F1 + IgG4 H4N4F1S1 + IgG4 H5N5F1 + IgG4 H5N4F1S1 + IgG4 H5N5F1S1 + IgG4 H5N4F1S2)
**IgG4 Galactosylation**	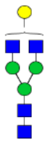	Galactosylation per antenna of diantennary glycans on IgG4	(1/2 * (IgG4 H4N4F1 + IgG4 H4N5F1 + IgG4 H4N4F1S1) + (IgG4 H5N4F1 + IgG4 H5N5F1 + IgG4 H5N4F1S1 + IgG4 H5N5F1S1 + IgG4 H5N4F1S2)) / (IgG4 H3N4F1 + IgG4 H4N4F1 + IgG4 H3N5F1 + IgG4 H5N4F1 + IgG4 H4N5F1 + IgG4 H4N4F1S1 + IgG4 H5N5F1 + IgG4 H5N4F1S1 + IgG4 H5N5F1S1 + IgG4 H5N4F1S2)
**IgG4 Sialylation**	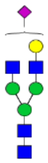	Sialylation per antenna of diantennary glycans on IgG4	(1/2 * (IgG4 H4N4F1S1 + IgG4 H5N4F1S1 + IgG4 H5N5F1S1) + (IgG4 H5N4F1S2)) / (IgG4 H3N4F1 + IgG4 H4N4F1 + IgG4 H3N5F1 + IgG4 H5N4F1 + IgG4 H4N5F1 + IgG4 H4N4F1S1 + IgG4 H5N5F1 + IgG4 H5N4F1S1 + IgG4 H5N5F1S1 + IgG4 H5N4F1S2)

**IgG4 Sialylation per galactose**	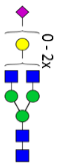	Sialylation per galactose of diantennary glycans on IgG4	IgG4 Sialylation / IgG4 Galactosylation
