# Supplementary material for: Differences in IgG Fc Glycosylation Are Associated with Outcome of Pediatric Meningococcal Sepsis
Source: mBio. 2018 Jun 19;9(3):e00546-18. doi: 10.1128/mBio.00546-18 (PMC6016251; doi:10.1128/mBio.00546-18)
Supplement: TABLE S3 [file mbo003183922st3.pdf]

	Age in healthy controls	
	Spearman's correlation coefficient (r)	*p* -value
**IgG1 Hybrid-type**	-0.77	**3.90E-10**
**IgG1 Bisection**	0.41	0.0048
**IgG1 Fucosylation**	-0.57	**3.70E-05**
**IgG1 Galactosylation**	-0.025	0.87
**IgG1 Sialylation**	-0.38	0.0096
**IgG1 Sialylation per galactose**	-0.58	**2.10E-05**
**IgG2/3 Hybrid-type**	-0.84	**5.00E-10**
**IgG2/3 Bisection**	0.51	**0.0022**
**IgG2/3 Fucosylation**	-0.32	0.067
**IgG2/3 Galactosylation**	-0.047	0.79
**IgG2/3 Sialylation**	-0.34	0.052
**IgG2/3 Sialylation per galactose**	-0.6	**0.00019**
**IgG4 Bisection**	-0.022	0.91
**IgG4 Galactosylation**	0.31	0.099
**IgG4 Sialylation**	0.066	0.73
**IgG4 Sialylation per galactose**	-0.42	0.023

α 0.0027
