# Supplementary material for: Differences in IgG Fc Glycosylation Are Associated with Outcome of Pediatric Meningococcal Sepsis
Source: mBio. 2018 Jun 19;9(3):e00546-18. doi: 10.1128/mBio.00546-18 (PMC6016251; doi:10.1128/mBio.00546-18)
Supplement: TABLE S4 [file mbo003183922st4.pdf]

	0 to 18 years old		
	**Healthy**	**Patients**	
	**Median % (IQR)**	**Median % (IQR)**	***p*-value**
**IgG1 Hybrid-type**	0.41 (0.34-0.46)	0.42 (0.35-0.49)	0.54
**IgG1 Bisection**	9.5 (8-11.6)	11.1 (9.6-13.1)	0.0044
**IgG1 Fucosylation**	97.8 (96.9-98.2)	96.1 (94.9-97.6)	**2.30E-05**
**IgG1 Galactosylation**	61.2 (56.6-63.7)	60.5 (57.2-62.7)	0.68
**IgG1 Sialylation**	10.6 (9.5-11.8)	10.4 (9.3-11.7)	0.67
**IgG1 Sialylation per galactose**	17.6 (16.1-18.6)	17.6 (16-18.6)	0.87
**IgG2/3 Hybrid-type**	0.36 (0.32-0.43)	0.35 (0.28-0.43)	0.72
**IgG2/3 Bisection**	8.8 (7.7-10.5)	9.6 (8.5-10.4)	0.14
**IgG2/3 Fucosylation**	98.3 (97.7-98.4)	97.8 (97.5-98.2)	0.043
**IgG2/3 Galactosylation**	52.7 (48.9-55.4)	51.8 (48.8-55.3)	0.92
**IgG2/3 Sialylation**	10.7 (9.2-12.9)	10.8 (9.7-11.9)	0.91
**IgG2/3 Sialylation per galactose**	20.9 (19.2-22.8)	20.5 (19.7-22.8)	0.87
**IgG4 Bisection**	12.7 (10.6-15.2)	14.5 (11.4-16.1)	0.27
**IgG4 Galactosylation**	53.0 (47.6-57.9)	54.3 (50.8-57.8)	0.34
**IgG4 Sialylation**	13.7 (11.5-14.7)	13.4 (12.1-15)	0.78
**IgG4 Sialylation per galactose**	25.1 (24-27)	24.7 (22.9-26.6)	0.47

	0 to 3.9 years old		
	**Healthy**	**Patients**	
	**Median % (IQR)**	**Median % (IQR)**	***p*-value**
**IgG1 Hybrid-type**	0.45 (0.43-0.52)	0.45 (0.41-0.53)	0.8
**IgG1 Bisection**	8.4 (7.4-10.3)	11.0 (9.2-12.9)	**0.002**
**IgG1 Fucosylation**	98.1 (97.8-98.4)	96.1 (94.2-97.3)	**1.90E-06**
**IgG1 Galactosylation**	61.9 (56.7-63.6)	61.6 (58.4-63.3)	0.82
**IgG1 Sialylation**	11.3 (10.1-12.8)	10.6 (9.7-11.9)	0.15
**IgG1 Sialylation per galactose**	18.4 (17.7-19.7)	17.8 (16.9-18.7)	0.037
**IgG2/3 Hybrid-type**	0.43 (0.40-0.54)	0.39 (0.30-0.49)	0.095
**IgG2/3 Bisection**	7.4 (6.3-8.1)	9.7 (8.8-11.2)	0.004
**IgG2/3 Fucosylation**	98.4 (98.3-98.7)	97.7 (97.5-98.1)	0.0057
**IgG2/3 Galactosylation**	54.5 (46.7-58.1)	51.4 (49.2-53.6)	0.51
**IgG2/3 Sialylation**	13.1 (9.3-14.3)	10.6 (9.7-11.7)	0.21
**IgG2/3 Sialylation per galactose**	23.7 (21.4-24.7)	21.1 (19.5-22.6)	0.014
**IgG4 Bisection**	13.2 (10.6-14.5)	15.5 (13.5-16)	0.17
**IgG4 Galactosylation**	51.6 (44.5-54.9)	54.5 (52.5-58.2)	0.07
**IgG4 Sialylation**	14.1 (11.3-14.9)	13.6 (12.4-14.7)	0.85
**IgG4 Sialylation per galactose**	26.5 (25.2-28.6)	24.9 (23-26.7)	0.15

	4 to 18 years old		
	**Healthy**	**Patients**	
	**Median % (IQR)**	**Median % (IQR)**	***p*-value**
**IgG1 Hybrid-type**	0.35 (0.31-0.38)	0.34 (0.31-0.40)	0.84
**IgG1 Bisection**	10.5 (9.2-11.9)	11.8 (10-13.1)	0.16
**IgG1 Fucosylation**	97.0 (96.1-97.5)	95.9 (95.5-97.4)	0.1
**IgG1 Galactosylation**	59.7 (55.2-64)	58.4 (56.8-62.5)	0.65
**IgG1 Sialylation**	9.8 (8.6-10.8)	9.5 (8.4-11)	0.99
**IgG1 Sialylation per galactose**	16.2 (15.1-17.4)	16.5 (15.1-17.8)	0.68
**IgG2/3 Hybrid-type**	0.33 (0.27-0.36)	0.31 (0.27-0.37)	0.88
**IgG2/3 Bisection**	9.5 (8.5-10.8)	9.6 (8.4-10.2)	0.86
**IgG2/3 Fucosylation**	98.0 (97.7-98.3)	98.0 (97.5-98.2)	0.67
**IgG2/3 Galactosylation**	52.5 (48.9-54.8)	54.5 (49.3-56.8)	0.31

**IgG2/3 Sialylation**	10.1 (9.2-11.4)	10.8 (9.5-12.8)	0.46
**IgG2/3 Sialylation per galactose**	20.4 (18.8-21.5)	20.2 (19.7-21.8)	0.63
**IgG4 Bisection**	12.7 (10.6-15.3)	12.0 (11.4-16.2)	0.88
**IgG4 Galactosylation**	53.5 (48.2-58.4)	54.1 (49.8-57.7)	1
**IgG4 Sialylation**	13.7 (11.8-14.7)	12.9 (11.1-14.7)	0.65
**IgG4 Sialylation per galactose**	24.6 (23.4-25.9)	24.6 (22.3-25.7)	0.67

α 0.0027
