# Supplementary material for: Differences in IgG Fc Glycosylation Are Associated with Outcome of Pediatric Meningococcal Sepsis
Source: mBio. 2018 Jun 19;9(3):e00546-18. doi: 10.1128/mBio.00546-18 (PMC6016251; doi:10.1128/mBio.00546-18)
Supplement: TABLE S5 [file mbo003183922st5.pdf]

| | Age in meningococcal patients (0 to 4 years old) | |
|---|---|---|
| | Spearman's correlation coefficient (r) | *p*-value |
| **IgG1 Hybrid-type** | 0.15 | 0.38 |
| **IgG1 Bisection** | -0.21 | 0.22 |
| **IgG1 Fucosylation** | 0.15 | 0.38 |
| **IgG1 Galactosylation** | 0.048 | 0.78 |
| **IgG1 Sialylation** | 0.25 | 0.14 |
| **IgG1 Sialylation per galactose** | 0.37 | 0.029 |
| **IgG2/3 Hybrid-type** | 0.26 | 0.15 |
| **IgG2/3 Bisection** | -0.35 | 0.052 |
| **IgG2/3 Fucosylation** | 0.014 | 0.94 |
| **IgG2/3 Galactosylation** | 0.078 | 0.67 |
| **IgG2/3 Sialylation** | 0.3 | 0.093 |
| **IgG2/3 Sialylation per galactose** | 0.41 | 0.019 |
| **IgG4 Bisection** | -0.15 | 0.49 |
| **IgG4 Galactosylation** | -0.012 | 0.96 |
| **IgG4 Sialylation** | 0.29 | 0.17 |
| **IgG4 Sialylation per galactose** | 0.44 | 0.034 |

| | Prism in meningococcal patients (0 to 4 years old) | |
|---|---|---|
| | Spearman's correlation coefficient (r) | *p*-value |
| **IgG1 Hybrid-type** | -0.42 | 0.013 |
| **IgG1 Bisection** | 0.14 | 0.44 |
| **IgG1 Fucosylation** | -0.25 | 0.15 |
| **IgG1 Galactosylation** | -0.26 | 0.13 |
| **IgG1 Sialylation** | -0.39 | 0.023 |
| **IgG1 Sialylation per galactose** | -0.45 | 0.0074 |
| **IgG2/3 Hybrid-type** | -0.33 | 0.073 |
| **IgG2/3 Bisection** | 0.39 | 0.031 |
| **IgG2/3 Fucosylation** | -0.18 | 0.32 |
| **IgG2/3 Galactosylation** | -0.34 | 0.061 |
| **IgG2/3 Sialylation** | -0.57 | **0.00092** |
| **IgG2/3 Sialylation per galactose** | -0.52 | 0.0029 |
| **IgG4 Bisection** | 0.17 | 0.44 |
| **IgG4 Galactosylation** | 0.11 | 0.63 |
| **IgG4 Sialylation** | -0.37 | 0.086 |
| **IgG4 Sialylation per galactose** | -0.62 | **0.0015** |

| | DIC in meningococcal patients (0 to 4 years old) | |
|---|---|---|
| | Spearman's correlation coefficient (r) | *p*-value |
| **IgG1 Hybrid-type** | -0.52 | 0.055 |
| **IgG1 Bisection** | 0.018 | 0.95 |
| **IgG1 Fucosylation** | -0.016 | 0.96 |
| **IgG1 Galactosylation** | -0.38 | 0.18 |
| **IgG1 Sialylation** | -0.46 | 0.1 |
| **IgG1 Sialylation per galactose** | -0.44 | 0.12 |
| **IgG2/3 Hybrid-type** | -0.53 | 0.091 |
| **IgG2/3 Bisection** | 0.27 | 0.42 |
| **IgG2/3 Fucosylation** | -0.22 | 0.53 |
| **IgG2/3 Galactosylation** | -0.21 | 0.54 |
| **IgG2/3 Sialylation** | -0.58 | 0.062 |
| **IgG2/3 Sialylation per galactose** | -0.65 | 0.032 |

| | | |
|---|---|---|
| **IgG4 Bisection** | 0.3 | 0.52 |
| **IgG4 Galactosylation** | -0.3 | 0.52 |
| **IgG4 Sialylation** | -0.52 | 0.23 |
| **IgG4 Sialylation per galactose** | -0.22 | 0.63 |

| | **RDAM in meningococcal patients (0 to 4 years old)** | |
|---|---|---|
| | **Spearman's correlation coefficient (r)** | ***p*-value** |
| **IgG1 Hybrid-type** | -0.56 | **5.00E-04** |
| **IgG1 Bisection** | 0.36 | 0.034 |
| **IgG1 Fucosylation** | -0.11 | 0.53 |
| **IgG1 Galactosylation** | -0.36 | 0.035 |
| **IgG1 Sialylation** | -0.5 | **0.0024** |
| **IgG1 Sialylation per galactose** | -0.47 | 0.0046 |
| **IgG2/3 Hybrid-type** | -0.41 | 0.022 |
| **IgG2/3 Bisection** | 0.36 | 0.05 |
| **IgG2/3 Fucosylation** | -0.024 | 0.9 |
| **IgG2/3 Galactosylation** | -0.16 | 0.38 |
| **IgG2/3 Sialylation** | -0.61 | **0.00025** |
| **IgG2/3 Sialylation per galactose** | -0.69 | **2.00E-05** |
| **IgG4 Bisection** | 0.3 | 0.16 |
| **IgG4 Galactosylation** | -0.24 | 0.28 |
| **IgG4 Sialylation** | -0.6 | 0.0032 |
| **IgG4 Sialylation per galactose** | -0.53 | 0.011 |

| | **Trombocytes in meningococcal patients (0 to 4 years** | |
|---|---|---|
| | **Spearman's correlation coefficient (r)** | ***p*-value** |
| **IgG1 Hybrid-type** | 0.55 | **0.00086** |
| **IgG1 Bisection** | -0.45 | 0.0075 |
| **IgG1 Fucosylation** | 0.37 | 0.033 |
| **IgG1 Galactosylation** | 0.29 | 0.092 |
| **IgG1 Sialylation** | 0.42 | 0.014 |
| **IgG1 Sialylation per galactose** | 0.44 | 0.0089 |
| **IgG2/3 Hybrid-type** | 0.55 | **0.0013** |
| **IgG2/3 Bisection** | -0.49 | 0.0053 |
| **IgG2/3 Fucosylation** | 0.18 | 0.33 |
| **IgG2/3 Galactosylation** | 0.095 | 0.61 |
| **IgG2/3 Sialylation** | 0.55 | **0.0013** |
| **IgG2/3 Sialylation per galactose** | 0.69 | **1.60E-05** |
| **IgG4 Bisection** | -0.38 | 0.077 |
| **IgG4 Galactosylation** | 0.12 | 0.58 |
| **IgG4 Sialylation** | 0.52 | 0.011 |
| **IgG4 Sialylation per galactose** | 0.49 | 0.018 |

| | **Fibrinogen in meningococcal patients (0 to 4 years old)** | |
|---|---|---|
| | **Spearman's correlation coefficient (r)** | ***p*-value** |
| **IgG1 Hybrid-type** | 0.56 | **0.0015** |
| **IgG1 Bisection** | -0.35 | 0.065 |
| **IgG1 Fucosylation** | 0.048 | 0.8 |
| **IgG1 Galactosylation** | 0.37 | 0.05 |
| **IgG1 Sialylation** | 0.43 | 0.021 |
| **IgG1 Sialylation per galactose** | 0.4 | 0.032 |
| **IgG2/3 Hybrid-type** | 0.56 | 0.0031 |

| | | |
|---|---|---|
| **IgG2/3 Bisection** | -0.45 | 0.02 |
| **IgG2/3 Fucosylation** | -0.015 | 0.94 |
| **IgG2/3 Galactosylation** | 0.14 | 0.51 |
| **IgG2/3 Sialylation** | 0.47 | 0.014 |
| **IgG2/3 Sialylation per galactose** | 0.6 | **0.0012** |
| **IgG4 Bisection** | -0.49 | 0.025 |
| **IgG4 Galactosylation** | 0.34 | 0.13 |
| **IgG4 Sialylation** | 0.64 | **0.0017** |
| **IgG4 Sialylation per galactose** | 0.64 | **0.0019** |

| | **PAI-1 in meningococcal patients (0 to 4 years old)** | |
|---|---|---|
| | **Spearman's correlation coefficient (r)** | ***p*-value** |
| **IgG1 Hybrid-type** | -0.44 | 0.055 |
| **IgG1 Bisection** | 0.21 | 0.37 |
| **IgG1 Fucosylation** | -0.52 | 0.021 |
| **IgG1 Galactosylation** | -0.22 | 0.36 |
| **IgG1 Sialylation** | -0.42 | 0.064 |
| **IgG1 Sialylation per galactose** | -0.39 | 0.088 |
| **IgG2/3 Hybrid-type** | -0.33 | 0.19 |
| **IgG2/3 Bisection** | 0.1 | 0.68 |
| **IgG2/3 Fucosylation** | -0.3 | 0.22 |
| **IgG2/3 Galactosylation** | 0.018 | 0.95 |
| **IgG2/3 Sialylation** | -0.72 | **0.001** |
| **IgG2/3 Sialylation per galactose** | -0.56 | 0.016 |
| **IgG4 Bisection** | 0.042 | 0.9 |
| **IgG4 Galactosylation** | -0.13 | 0.68 |
| **IgG4 Sialylation** | -0.32 | 0.31 |
| **IgG4 Sialylation per galactose** | -0.39 | 0.21 |

| | **CRP in meningococcal patients (0 to 4 years old)** | |
|---|---|---|
| | **Spearman's correlation coefficient (r)** | ***p*-value** |
| **IgG1 Hybrid-type** | 0.4 | 0.019 |
| **IgG1 Bisection** | -0.036 | 0.84 |
| **IgG1 Fucosylation** | -0.021 | 0.91 |
| **IgG1 Galactosylation** | 0.28 | 0.11 |
| **IgG1 Sialylation** | 0.25 | 0.15 |
| **IgG1 Sialylation per galactose** | 0.18 | 0.32 |
| **IgG2/3 Hybrid-type** | 0.39 | 0.032 |
| **IgG2/3 Bisection** | -0.098 | 0.6 |
| **IgG2/3 Fucosylation** | 0.019 | 0.92 |
| **IgG2/3 Galactosylation** | 0.11 | 0.55 |
| **IgG2/3 Sialylation** | 0.22 | 0.24 |
| **IgG2/3 Sialylation per galactose** | 0.28 | 0.13 |
| **IgG4 Bisection** | -0.23 | 0.29 |
| **IgG4 Galactosylation** | 0.35 | 0.1 |
| **IgG4 Sialylation** | 0.38 | 0.072 |
| **IgG4 Sialylation per galactose** | 0.18 | 0.42 |

| | **Leukocytes in meningococcal patients (0 to 4 years old)** | |
|---|---|---|
| | **Spearman's correlation coefficient (r)** | ***p*-value** |
| **IgG1 Hybrid-type** | 0.4 | 0.018 |
| **IgG1 Bisection** | -0.19 | 0.29 |

| | | |
|---|---|---|
| **IgG1 Fucosylation** | -0.072 | 0.68 |
| **IgG1 Galactosylation** | 0.27 | 0.12 |
| **IgG1 Sialylation** | 0.34 | 0.052 |
| **IgG1 Sialylation per galactose** | 0.33 | 0.057 |
| **IgG2/3 Hybrid-type** | 0.21 | 0.25 |
| **IgG2/3 Bisection** | -0.19 | 0.29 |
| **IgG2/3 Fucosylation** | -0.077 | 0.68 |
| **IgG2/3 Galactosylation** | 0.14 | 0.45 |
| **IgG2/3 Sialylation** | 0.38 | 0.037 |
| **IgG2/3 Sialylation per galactose** | 0.38 | 0.037 |
| **IgG4 Bisection** | -0.3 | 0.17 |
| **IgG4 Galactosylation** | 0.14 | 0.53 |
| **IgG4 Sialylation** | 0.38 | 0.075 |
| **IgG4 Sialylation per galactose** | 0.37 | 0.08 |

| | **PCT in meningococcal patients (0 to 4 years old)** | |
|---|---|---|
| | **Spearman's correlation coefficient (r)** | ***p*-value** |
| **IgG1 Hybrid-type** | -0.067 | 0.76 |
| **IgG1 Bisection** | 0.044 | 0.84 |
| **IgG1 Fucosylation** | -0.43 | 0.043 |
| **IgG1 Galactosylation** | 0.27 | 0.2 |
| **IgG1 Sialylation** | 0.23 | 0.29 |
| **IgG1 Sialylation per galactose** | 0.059 | 0.79 |
| **IgG2/3 Hybrid-type** | -0.18 | 0.45 |
| **IgG2/3 Bisection** | 0.24 | 0.29 |
| **IgG2/3 Fucosylation** | -0.4 | 0.072 |
| **IgG2/3 Galactosylation** | 0.34 | 0.13 |
| **IgG2/3 Sialylation** | 0.14 | 0.55 |
| **IgG2/3 Sialylation per galactose** | -0.12 | 0.61 |
| **IgG4 Bisection** | 0.021 | 0.94 |
| **IgG4 Galactosylation** | 0.52 | 0.049 |
| **IgG4 Sialylation** | 0.082 | 0.77 |
| **IgG4 Sialylation per galactose** | -0.27 | 0.32 |

| | **TNF in meningococcal patients (0 to 4 years old)** | |
|---|---|---|
| | **Spearman's correlation coefficient (r)** | ***p*-value** |
| **IgG1 Hybrid-type** | -0.41 | 0.058 |
| **IgG1 Bisection** | 0.17 | 0.44 |
| **IgG1 Fucosylation** | -0.46 | 0.033 |
| **IgG1 Galactosylation** | 0.083 | 0.71 |
| **IgG1 Sialylation** | -0.15 | 0.51 |
| **IgG1 Sialylation per galactose** | -0.21 | 0.34 |
| **IgG2/3 Hybrid-type** | -0.48 | 0.034 |
| **IgG2/3 Bisection** | 0.071 | 0.76 |
| **IgG2/3 Fucosylation** | -0.37 | 0.11 |
| **IgG2/3 Galactosylation** | -0.084 | 0.73 |
| **IgG2/3 Sialylation** | -0.47 | 0.036 |
| **IgG2/3 Sialylation per galactose** | -0.42 | 0.066 |
| **IgG4 Bisection** | 0.23 | 0.46 |
| **IgG4 Galactosylation** | 0.35 | 0.25 |
| **IgG4 Sialylation** | -0.23 | 0.46 |
| **IgG4 Sialylation per galactose** | -0.58 | 0.04 |

| | IL6 in meningococcal patients (0 to 4 years old) | |
|---|---|---|
| | Spearman's correlation coefficient (r) | *p*-value |
| **IgG1 Hybrid-type** | -0.53 | 0.018 |
| **IgG1 Bisection** | 0.3 | 0.2 |
| **IgG1 Fucosylation** | -0.65 | **0.0023** |
| **IgG1 Galactosylation** | -0.23 | 0.33 |
| **IgG1 Sialylation** | -0.41 | 0.076 |
| **IgG1 Sialylation per galactose** | -0.34 | 0.14 |
| **IgG2/3 Hybrid-type** | -0.57 | 0.014 |
| **IgG2/3 Bisection** | 0.23 | 0.36 |
| **IgG2/3 Fucosylation** | -0.33 | 0.18 |
| **IgG2/3 Galactosylation** | 0.09 | 0.72 |
| **IgG2/3 Sialylation** | -0.62 | 0.0074 |
| **IgG2/3 Sialylation per galactose** | -0.54 | 0.023 |
| **IgG4 Bisection** | 0.091 | 0.78 |
| **IgG4 Galactosylation** | 0.035 | 0.92 |
| **IgG4 Sialylation** | -0.29 | 0.35 |
| **IgG4 Sialylation per galactose** | -0.75 | 0.0074 |

| | IL8 in meningococcal patients (0 to 4 years old) | |
|---|---|---|
| | Spearman's correlation coefficient (r) | *p*-value |
| **IgG1 Hybrid-type** | -0.55 | 0.013 |
| **IgG1 Bisection** | 0.26 | 0.27 |
| **IgG1 Fucosylation** | -0.58 | 0.0086 |
| **IgG1 Galactosylation** | -0.22 | 0.36 |
| **IgG1 Sialylation** | -0.34 | 0.14 |
| **IgG1 Sialylation per galactose** | -0.27 | 0.25 |
| **IgG2/3 Hybrid-type** | -0.49 | 0.043 |
| **IgG2/3 Bisection** | 0.17 | 0.49 |
| **IgG2/3 Fucosylation** | -0.27 | 0.27 |
| **IgG2/3 Galactosylation** | 0.09 | 0.72 |
| **IgG2/3 Sialylation** | -0.56 | 0.018 |
| **IgG2/3 Sialylation per galactose** | -0.48 | 0.046 |
| **IgG4 Bisection** | 0.035 | 0.92 |
| **IgG4 Galactosylation** | 0.11 | 0.73 |
| **IgG4 Sialylation** | -0.15 | 0.64 |
| **IgG4 Sialylation per galactose** | -0.62 | 0.037 |

| | Age in meningococcal patients (0 to 18 years old) | |
|---|---|---|
| | Spearman's correlation coefficient (r) | *p*-value |
| **IgG1 Hybrid-type** | -0.52 | **4.30E-05** |
| **IgG1 Bisection** | 0.089 | 0.51 |
| **IgG1 Fucosylation** | 0.11 | 0.42 |
| **IgG1 Galactosylation** | -0.2 | 0.13 |
| **IgG1 Sialylation** | -0.22 | 0.094 |
| **IgG1 Sialylation per galactose** | -0.2 | 0.13 |
| **IgG2/3 Hybrid-type** | -0.19 | 0.17 |
| **IgG2/3 Bisection** | -0.09 | 0.52 |
| **IgG2/3 Fucosylation** | 0.072 | 0.6 |
| **IgG2/3 Galactosylation** | 0.17 | 0.22 |
| **IgG2/3 Sialylation** | 0.081 | 0.56 |
| **IgG2/3 Sialylation per galactose** | -0.033 | 0.81 |
| **IgG4 Bisection** | -0.27 | 0.078 |
| **IgG4 Galactosylation** | -0.097 | 0.52 |
| **IgG4 Sialylation** | -0.1 | 0.51 |
| **IgG4 Sialylation per galactose** | -0.077 | 0.61 |

| | Prism in meningococcal patients (0 to 18 years old) | |
|---|---|---|
| | Spearman's correlation coefficient (r) | *p*-value |
| **IgG1 Hybrid-type** | -0.09 | 0.5 |
| **IgG1 Bisection** | -0.021 | 0.88 |
| **IgG1 Fucosylation** | -0.15 | 0.26 |
| **IgG1 Galactosylation** | -0.022 | 0.87 |
| **IgG1 Sialylation** | -0.13 | 0.34 |
| **IgG1 Sialylation per galactose** | -0.2 | 0.13 |
| **IgG2/3 Hybrid-type** | -0.2 | 0.14 |
| **IgG2/3 Bisection** | 0.16 | 0.26 |
| **IgG2/3 Fucosylation** | -0.29 | 0.033 |
| **IgG2/3 Galactosylation** | -0.16 | 0.26 |
| **IgG2/3 Sialylation** | -0.28 | 0.037 |
| **IgG2/3 Sialylation per galactose** | -0.27 | 0.043 |
| **IgG4 Bisection** | -0.0066 | 0.97 |
| **IgG4 Galactosylation** | 0.12 | 0.44 |
| **IgG4 Sialylation** | -0.061 | 0.68 |
| **IgG4 Sialylation per galactose** | -0.32 | 0.028 |

| | DIC in meningococcal patients (0 to 18 years old) | |
|---|---|---|
| | Spearman's correlation coefficient (r) | *p*-value |
| **IgG1 Hybrid-type** | -0.19 | 0.4 |
| **IgG1 Bisection** | -0.0029 | 0.99 |
| **IgG1 Fucosylation** | -0.037 | 0.87 |
| **IgG1 Galactosylation** | -0.19 | 0.41 |
| **IgG1 Sialylation** | -0.26 | 0.24 |
| **IgG1 Sialylation per galactose** | -0.27 | 0.23 |
| **IgG2/3 Hybrid-type** | -0.42 | 0.073 |
| **IgG2/3 Bisection** | -0.12 | 0.61 |
| **IgG2/3 Fucosylation** | -0.31 | 0.19 |
| **IgG2/3 Galactosylation** | -0.15 | 0.54 |
| **IgG2/3 Sialylation** | -0.41 | 0.084 |
| **IgG2/3 Sialylation per galactose** | -0.56 | 0.013 |

| | | |
|---|---|---|
| **IgG4 Bisection** | 0.14 | 0.64 |
| **IgG4 Galactosylation** | -0.21 | 0.47 |
| **IgG4 Sialylation** | -0.27 | 0.35 |
| **IgG4 Sialylation per galactose** | -0.19 | 0.51 |

| | **RDAM in meningococcal patients (0 to 18 years old)** | |
|---|---|---|
| | **Spearman's correlation coefficient (r)** | ***p*-value** |
| **IgG1 Hybrid-type** | -0.19 | 0.15 |
| **IgG1 Bisection** | 0.26 | 0.052 |
| **IgG1 Fucosylation** | -0.15 | 0.27 |
| **IgG1 Galactosylation** | -0.18 | 0.19 |
| **IgG1 Sialylation** | -0.3 | 0.023 |
| **IgG1 Sialylation per galactose** | -0.28 | 0.037 |
| **IgG2/3 Hybrid-type** | -0.3 | 0.029 |
| **IgG2/3 Bisection** | 0.23 | 0.098 |
| **IgG2/3 Fucosylation** | -0.18 | 0.19 |
| **IgG2/3 Galactosylation** | -0.11 | 0.44 |
| **IgG2/3 Sialylation** | -0.34 | 0.011 |
| **IgG2/3 Sialylation per galactose** | -0.43 | **0.0012** |
| **IgG4 Bisection** | 0.094 | 0.54 |
| **IgG4 Galactosylation** | 0.036 | 0.81 |
| **IgG4 Sialylation** | -0.21 | 0.18 |
| **IgG4 Sialylation per galactose** | -0.37 | 0.013 |

| | **Trombocytes in meningococcal patients (0 to 18 years** | |
|---|---|---|
| | **Spearman's correlation coefficient (r)** | ***p*-value** |
| **IgG1 Hybrid-type** | 0.28 | 0.031 |
| **IgG1 Bisection** | -0.34 | 0.0088 |
| **IgG1 Fucosylation** | 0.35 | 0.0063 |
| **IgG1 Galactosylation** | 0.095 | 0.48 |
| **IgG1 Sialylation** | 0.31 | 0.017 |
| **IgG1 Sialylation per galactose** | 0.38 | 0.0033 |
| **IgG2/3 Hybrid-type** | 0.42 | **0.0016** |
| **IgG2/3 Bisection** | -0.29 | 0.033 |
| **IgG2/3 Fucosylation** | 0.3 | 0.024 |
| **IgG2/3 Galactosylation** | 0.041 | 0.76 |
| **IgG2/3 Sialylation** | 0.36 | 0.0067 |
| **IgG2/3 Sialylation per galactose** | 0.51 | **6.10E-05** |
| **IgG4 Bisection** | -0.095 | 0.53 |
| **IgG4 Galactosylation** | -0.13 | 0.41 |
| **IgG4 Sialylation** | 0.21 | 0.16 |
| **IgG4 Sialylation per galactose** | 0.43 | 0.0027 |

| | **Fibrinogen in meningococcal patients (0 to 18 years** | |
|---|---|---|
| | **Spearman's correlation coefficient (r)** | ***p*-value** |
| **IgG1 Hybrid-type** | 0.42 | **0.0021** |
| **IgG1 Bisection** | -0.23 | 0.094 |
| **IgG1 Fucosylation** | 0.11 | 0.46 |
| **IgG1 Galactosylation** | 0.17 | 0.24 |
| **IgG1 Sialylation** | 0.34 | 0.013 |
| **IgG1 Sialylation per galactose** | 0.35 | 0.011 |
| **IgG2/3 Hybrid-type** | 0.52 | **0.00011** |

| | | |
|---|---|---|
| **IgG2/3 Bisection** | -0.25 | 0.081 |
| **IgG2/3 Fucosylation** | 0.11 | 0.47 |
| **IgG2/3 Galactosylation** | -0.039 | 0.79 |
| **IgG2/3 Sialylation** | 0.22 | 0.13 |
| **IgG2/3 Sialylation per galactose** | 0.39 | 0.0059 |
| **IgG4 Bisection** | -0.054 | 0.73 |
| **IgG4 Galactosylation** | 0.0086 | 0.96 |
| **IgG4 Sialylation** | 0.26 | 0.098 |
| **IgG4 Sialylation per galactose** | 0.46 | **0.0021** |

| | **PAI-1 in meningococcal patients (0 to 18 years old)** | |
|---|---|---|
| | **Spearman's correlation coefficient (r)** | ***p*-value** |
| **IgG1 Hybrid-type** | 0.0071 | 0.97 |
| **IgG1 Bisection** | -0.16 | 0.37 |
| **IgG1 Fucosylation** | -0.2 | 0.24 |
| **IgG1 Galactosylation** | 0.1 | 0.57 |
| **IgG1 Sialylation** | -0.14 | 0.42 |
| **IgG1 Sialylation per galactose** | -0.2 | 0.24 |
| **IgG2/3 Hybrid-type** | -0.17 | 0.35 |
| **IgG2/3 Bisection** | -0.12 | 0.51 |
| **IgG2/3 Fucosylation** | -0.12 | 0.5 |
| **IgG2/3 Galactosylation** | 0.17 | 0.36 |
| **IgG2/3 Sialylation** | -0.15 | 0.41 |
| **IgG2/3 Sialylation per galactose** | -0.29 | 0.1 |
| **IgG4 Bisection** | -0.036 | 0.86 |
| **IgG4 Galactosylation** | 0.035 | 0.87 |
| **IgG4 Sialylation** | -0.079 | 0.7 |
| **IgG4 Sialylation per galactose** | -0.24 | 0.25 |

| | **CRP in meningococcal patients (0 to 18 years old)** | |
|---|---|---|
| | **Spearman's correlation coefficient (r)** | ***p*-value** |
| **IgG1 Hybrid-type** | 0.16 | 0.25 |
| **IgG1 Bisection** | -0.045 | 0.74 |
| **IgG1 Fucosylation** | 0.042 | 0.76 |
| **IgG1 Galactosylation** | 0.25 | 0.065 |
| **IgG1 Sialylation** | 0.23 | 0.091 |
| **IgG1 Sialylation per galactose** | 0.14 | 0.31 |
| **IgG2/3 Hybrid-type** | 0.36 | 0.0089 |
| **IgG2/3 Bisection** | -0.072 | 0.61 |
| **IgG2/3 Fucosylation** | 0.053 | 0.71 |
| **IgG2/3 Galactosylation** | 0.18 | 0.2 |
| **IgG2/3 Sialylation** | 0.24 | 0.089 |
| **IgG2/3 Sialylation per galactose** | 0.23 | 0.092 |
| **IgG4 Bisection** | -0.14 | 0.37 |
| **IgG4 Galactosylation** | 0.22 | 0.15 |
| **IgG4 Sialylation** | 0.21 | 0.17 |
| **IgG4 Sialylation per galactose** | 0.21 | 0.18 |

| | **Leukocytes in meningococcal patients (0 to 18 years** | |
|---|---|---|
| | **Spearman's correlation coefficient (r)** | ***p*-value** |
| **IgG1 Hybrid-type** | 0.27 | 0.041 |
| **IgG1 Bisection** | -0.12 | 0.35 |

| | | |
|---|---|---|
| **IgG1 Fucosylation** | 0.019 | 0.88 |
| **IgG1 Galactosylation** | 0.22 | 0.099 |
| **IgG1 Sialylation** | 0.31 | 0.019 |
| **IgG1 Sialylation per galactose** | 0.27 | 0.042 |
| **IgG2/3 Hybrid-type** | 0.3 | 0.026 |
| **IgG2/3 Bisection** | -0.066 | 0.63 |
| **IgG2/3 Fucosylation** | 0.059 | 0.67 |
| **IgG2/3 Galactosylation** | 0.14 | 0.32 |
| **IgG2/3 Sialylation** | 0.25 | 0.071 |
| **IgG2/3 Sialylation per galactose** | 0.25 | 0.069 |
| **IgG4 Bisection** | -0.0073 | 0.96 |
| **IgG4 Galactosylation** | -0.027 | 0.86 |
| **IgG4 Sialylation** | 0.14 | 0.34 |
| **IgG4 Sialylation per galactose** | 0.27 | 0.073 |

| | **PCT in meningococcal patients (0 to 18 years old)** | |
|---|---|---|
| | **Spearman's correlation coefficient (r)** | ***p*-value** |
| **IgG1 Hybrid-type** | 0.11 | 0.49 |
| **IgG1 Bisection** | -0.13 | 0.44 |
| **IgG1 Fucosylation** | -0.1 | 0.53 |
| **IgG1 Galactosylation** | 0.23 | 0.17 |
| **IgG1 Sialylation** | 0.14 | 0.39 |
| **IgG1 Sialylation per galactose** | 0.045 | 0.79 |
| **IgG2/3 Hybrid-type** | -0.038 | 0.83 |
| **IgG2/3 Bisection** | -0.034 | 0.84 |
| **IgG2/3 Fucosylation** | -0.15 | 0.38 |
| **IgG2/3 Galactosylation** | 0.16 | 0.36 |
| **IgG2/3 Sialylation** | 0.12 | 0.49 |
| **IgG2/3 Sialylation per galactose** | -0.016 | 0.93 |
| **IgG4 Bisection** | 0.0025 | 0.99 |
| **IgG4 Galactosylation** | 0.03 | 0.88 |
| **IgG4 Sialylation** | -0.082 | 0.67 |
| **IgG4 Sialylation per galactose** | -0.12 | 0.52 |

| | **TNF in meningococcal patients (0 to 18 years old)** | |
|---|---|---|
| | **Spearman's correlation coefficient (r)** | ***p*-value** |
| **IgG1 Hybrid-type** | -0.07 | 0.67 |
| **IgG1 Bisection** | -0.053 | 0.74 |
| **IgG1 Fucosylation** | -0.24 | 0.14 |
| **IgG1 Galactosylation** | -0.0087 | 0.96 |
| **IgG1 Sialylation** | -0.14 | 0.38 |
| **IgG1 Sialylation per galactose** | -0.1 | 0.54 |
| **IgG2/3 Hybrid-type** | -0.23 | 0.17 |
| **IgG2/3 Bisection** | -0.079 | 0.64 |
| **IgG2/3 Fucosylation** | -0.22 | 0.18 |
| **IgG2/3 Galactosylation** | -0.081 | 0.63 |
| **IgG2/3 Sialylation** | -0.22 | 0.19 |
| **IgG2/3 Sialylation per galactose** | -0.22 | 0.18 |
| **IgG4 Bisection** | 0.063 | 0.74 |
| **IgG4 Galactosylation** | 0.033 | 0.86 |
| **IgG4 Sialylation** | -0.24 | 0.2 |
| **IgG4 Sialylation per galactose** | -0.36 | 0.05 |

| | IL6 in meningococcal patients (0 to 18 years old) | |
|---|---|---|
| | Spearman's correlation coefficient (r) | *p* -value |
| **IgG1 Hybrid-type** | -0.081 | 0.64 |
| **IgG1 Bisection** | -0.084 | 0.63 |
| **IgG1 Fucosylation** | -0.31 | 0.073 |
| **IgG1 Galactosylation** | 0.051 | 0.77 |
| **IgG1 Sialylation** | -0.19 | 0.26 |
| **IgG1 Sialylation per galactose** | -0.2 | 0.24 |
| **IgG2/3 Hybrid-type** | -0.29 | 0.11 |
| **IgG2/3 Bisection** | -0.097 | 0.59 |
| **IgG2/3 Fucosylation** | -0.17 | 0.35 |
| **IgG2/3 Galactosylation** | 0.11 | 0.53 |
| **IgG2/3 Sialylation** | -0.19 | 0.28 |
| **IgG2/3 Sialylation per galactose** | -0.31 | 0.077 |
| **IgG4 Bisection** | -0.084 | 0.68 |
| **IgG4 Galactosylation** | -0.019 | 0.93 |
| **IgG4 Sialylation** | -0.22 | 0.29 |
| **IgG4 Sialylation per galactose** | -0.39 | 0.047 |

| | IL8 in meningococcal patients (0 to 18 years old) | |
|---|---|---|
| | Spearman's correlation coefficient (r) | *p* -value |
| **IgG1 Hybrid-type** | -0.14 | 0.43 |
| **IgG1 Bisection** | -0.12 | 0.49 |
| **IgG1 Fucosylation** | -0.32 | 0.064 |
| **IgG1 Galactosylation** | 0.033 | 0.85 |
| **IgG1 Sialylation** | -0.2 | 0.26 |
| **IgG1 Sialylation per galactose** | -0.17 | 0.33 |
| **IgG2/3 Hybrid-type** | -0.24 | 0.18 |
| **IgG2/3 Bisection** | -0.12 | 0.5 |
| **IgG2/3 Fucosylation** | -0.21 | 0.24 |
| **IgG2/3 Galactosylation** | 0.088 | 0.63 |
| **IgG2/3 Sialylation** | -0.19 | 0.28 |
| **IgG2/3 Sialylation per galactose** | -0.29 | 0.096 |
| **IgG4 Bisection** | -0.23 | 0.25 |
| **IgG4 Galactosylation** | 0.042 | 0.84 |
| **IgG4 Sialylation** | -0.15 | 0.48 |
| **IgG4 Sialylation per galactose** | -0.4 | 0.043 |

| | Age in meningococcal patients (4 to 18 years old) | |
|---|---|---|
| | Spearman's correlation coefficient (r) | *p* -value |
| **IgG1 Hybrid-type** | -0.61 | 0.0032 |
| **IgG1 Bisection** | 0.39 | 0.072 |
| **IgG1 Fucosylation** | -0.63 | **0.002** |
| **IgG1 Galactosylation** | -0.4 | 0.067 |
| **IgG1 Sialylation** | -0.44 | 0.044 |
| **IgG1 Sialylation per galactose** | -0.35 | 0.11 |
| **IgG2/3 Hybrid-type** | -0.36 | 0.1 |
| **IgG2/3 Bisection** | 0.35 | 0.11 |
| **IgG2/3 Fucosylation** | -0.22 | 0.33 |
| **IgG2/3 Galactosylation** | -0.11 | 0.62 |
| **IgG2/3 Sialylation** | -0.22 | 0.32 |
| **IgG2/3 Sialylation per galactose** | -0.19 | 0.39 |
| **IgG4 Bisection** | -0.23 | 0.31 |
| **IgG4 Galactosylation** | 0.11 | 0.64 |
| **IgG4 Sialylation** | -0.088 | 0.7 |
| **IgG4 Sialylation per galactose** | -0.29 | 0.21 |

| | Prism in meningococcal patients (4 to 18 years old) | |
|---|---|---|
| | Spearman's correlation coefficient (r) | *p* -value |
| **IgG1 Hybrid-type** | -0.0073 | 0.97 |
| **IgG1 Bisection** | -0.22 | 0.33 |
| **IgG1 Fucosylation** | 0.23 | 0.3 |
| **IgG1 Galactosylation** | 0.2 | 0.37 |
| **IgG1 Sialylation** | 0.1 | 0.66 |
| **IgG1 Sialylation per galactose** | -0.02 | 0.93 |
| **IgG2/3 Hybrid-type** | -0.21 | 0.35 |
| **IgG2/3 Bisection** | -0.12 | 0.58 |
| **IgG2/3 Fucosylation** | -0.26 | 0.24 |
| **IgG2/3 Galactosylation** | 0.046 | 0.84 |
| **IgG2/3 Sialylation** | 0.1 | 0.66 |
| **IgG2/3 Sialylation per galactose** | -0.041 | 0.86 |
| **IgG4 Bisection** | 0.055 | 0.81 |
| **IgG4 Galactosylation** | 0.047 | 0.84 |
| **IgG4 Sialylation** | -0.013 | 0.96 |
| **IgG4 Sialylation per galactose** | -0.11 | 0.64 |

| | DIC in meningococcal patients (4 to 18 years old) | |
|---|---|---|
| | Spearman's correlation coefficient (r) | *p* -value |
| **IgG1 Hybrid-type** | 0.024 | 0.95 |
| **IgG1 Bisection** | 0 | 1 |
| **IgG1 Fucosylation** | -0.012 | 0.98 |
| **IgG1 Galactosylation** | 0.2 | 0.63 |
| **IgG1 Sialylation** | -0.14 | 0.73 |
| **IgG1 Sialylation per galactose** | -0.14 | 0.73 |
| **IgG2/3 Hybrid-type** | -0.46 | 0.25 |
| **IgG2/3 Bisection** | -0.42 | 0.3 |
| **IgG2/3 Fucosylation** | -0.54 | 0.17 |
| **IgG2/3 Galactosylation** | 0 | 1 |
| **IgG2/3 Sialylation** | -0.3 | 0.47 |
| **IgG2/3 Sialylation per galactose** | -0.46 | 0.25 |

| | | |
|---|---|---|
| **IgG4 Bisection** | -0.09 | 0.85 |
| **IgG4 Galactosylation** | -0.2 | 0.67 |
| **IgG4 Sialylation** | -0.18 | 0.7 |
| **IgG4 Sialylation per galactose** | -0.31 | 0.5 |

| | **RDAM in meningococcal patients (4 to 18 years old)** | |
|---|---|---|
| | **Spearman's correlation coefficient (r)** | ***p*-value** |
| **IgG1 Hybrid-type** | -0.14 | 0.55 |
| **IgG1 Bisection** | 0.026 | 0.91 |
| **IgG1 Fucosylation** | -0.04 | 0.86 |
| **IgG1 Galactosylation** | 0.15 | 0.51 |
| **IgG1 Sialylation** | -0.082 | 0.72 |
| **IgG1 Sialylation per galactose** | -0.16 | 0.49 |
| **IgG2/3 Hybrid-type** | -0.4 | 0.073 |
| **IgG2/3 Bisection** | -0.12 | 0.59 |
| **IgG2/3 Fucosylation** | -0.32 | 0.16 |
| **IgG2/3 Galactosylation** | 0.14 | 0.54 |
| **IgG2/3 Sialylation** | 0.058 | 0.8 |
| **IgG2/3 Sialylation per galactose** | -0.18 | 0.43 |
| **IgG4 Bisection** | 0.053 | 0.83 |
| **IgG4 Galactosylation** | 0.26 | 0.26 |
| **IgG4 Sialylation** | -0.0045 | 0.99 |
| **IgG4 Sialylation per galactose** | -0.28 | 0.23 |

| | **Trombocytes in meningococcal patients (4 to 18 years** | |
|---|---|---|
| | **Spearman's correlation coefficient (r)** | ***p*-value** |
| **IgG1 Hybrid-type** | 0.27 | 0.23 |
| **IgG1 Bisection** | -0.019 | 0.94 |
| **IgG1 Fucosylation** | 0.088 | 0.7 |
| **IgG1 Galactosylation** | -0.11 | 0.62 |
| **IgG1 Sialylation** | 0.16 | 0.48 |
| **IgG1 Sialylation per galactose** | 0.27 | 0.22 |
| **IgG2/3 Hybrid-type** | 0.35 | 0.12 |
| **IgG2/3 Bisection** | 0.17 | 0.46 |
| **IgG2/3 Fucosylation** | 0.3 | 0.18 |
| **IgG2/3 Galactosylation** | -0.013 | 0.96 |
| **IgG2/3 Sialylation** | 0.043 | 0.85 |
| **IgG2/3 Sialylation per galactose** | 0.25 | 0.26 |
| **IgG4 Bisection** | 0.062 | 0.79 |
| **IgG4 Galactosylation** | -0.25 | 0.27 |
| **IgG4 Sialylation** | -0.017 | 0.94 |
| **IgG4 Sialylation per galactose** | 0.3 | 0.19 |

| | **Fibrinogen in meningococcal patients (4 to 18 years** | |
|---|---|---|
| | **Spearman's correlation coefficient (r)** | ***p*-value** |
| **IgG1 Hybrid-type** | 0.39 | 0.085 |
| **IgG1 Bisection** | 0.12 | 0.59 |
| **IgG1 Fucosylation** | 0.13 | 0.57 |
| **IgG1 Galactosylation** | -0.16 | 0.48 |
| **IgG1 Sialylation** | 0.14 | 0.54 |
| **IgG1 Sialylation per galactose** | 0.27 | 0.23 |
| **IgG2/3 Hybrid-type** | 0.37 | 0.097 |

| | | |
|---|---|---|
| **IgG2/3 Bisection** | 0.15 | 0.51 |
| **IgG2/3 Fucosylation** | 0.29 | 0.2 |
| **IgG2/3 Galactosylation** | -0.28 | 0.23 |
| **IgG2/3 Sialylation** | -0.19 | 0.41 |
| **IgG2/3 Sialylation per galactose** | 0.088 | 0.7 |
| **IgG4 Bisection** | 0.22 | 0.36 |
| **IgG4 Galactosylation** | -0.38 | 0.094 |
| **IgG4 Sialylation** | -0.064 | 0.79 |
| **IgG4 Sialylation per galactose** | 0.28 | 0.23 |

| | **PAI-1 in meningococcal patients (4 to 18 years old)** | |
|---|---|---|
| | **Spearman's correlation coefficient (r)** | ***p*-value** |
| **IgG1 Hybrid-type** | -0.11 | 0.7 |
| **IgG1 Bisection** | -0.7 | 0.0046 |
| **IgG1 Fucosylation** | 0.39 | 0.16 |
| **IgG1 Galactosylation** | 0.29 | 0.29 |
| **IgG1 Sialylation** | 0.1 | 0.71 |
| **IgG1 Sialylation per galactose** | -0.096 | 0.73 |
| **IgG2/3 Hybrid-type** | -0.046 | 0.87 |
| **IgG2/3 Bisection** | -0.42 | 0.12 |
| **IgG2/3 Fucosylation** | -0.064 | 0.82 |
| **IgG2/3 Galactosylation** | 0.41 | 0.13 |
| **IgG2/3 Sialylation** | 0.35 | 0.2 |
| **IgG2/3 Sialylation per galactose** | 0.064 | 0.82 |
| **IgG4 Bisection** | -0.27 | 0.34 |
| **IgG4 Galactosylation** | 0.15 | 0.61 |
| **IgG4 Sialylation** | 0.064 | 0.83 |
| **IgG4 Sialylation per galactose** | -0.024 | 0.94 |

| | **CRP in meningococcal patients (4 to 18 years old)** | |
|---|---|---|
| | **Spearman's correlation coefficient (r)** | ***p*-value** |
| **IgG1 Hybrid-type** | 0.37 | 0.11 |
| **IgG1 Bisection** | -0.067 | 0.78 |
| **IgG1 Fucosylation** | 0.085 | 0.72 |
| **IgG1 Galactosylation** | 0.15 | 0.54 |
| **IgG1 Sialylation** | 0.36 | 0.12 |
| **IgG1 Sialylation per galactose** | 0.42 | 0.064 |
| **IgG2/3 Hybrid-type** | 0.68 | **0.00092** |
| **IgG2/3 Bisection** | 0.047 | 0.85 |
| **IgG2/3 Fucosylation** | 0.065 | 0.79 |
| **IgG2/3 Galactosylation** | -0.009 | 0.97 |
| **IgG2/3 Sialylation** | 0.14 | 0.57 |
| **IgG2/3 Sialylation per galactose** | 0.36 | 0.12 |
| **IgG4 Bisection** | -0.3 | 0.21 |
| **IgG4 Galactosylation** | 0.015 | 0.95 |
| **IgG4 Sialylation** | 0.35 | 0.14 |
| **IgG4 Sialylation per galactose** | 0.46 | 0.048 |

| | **Leukocytes in meningococcal patients (4 to 18 years** | |
|---|---|---|
| | **Spearman's correlation coefficient (r)** | ***p*-value** |
| **IgG1 Hybrid-type** | 0.61 | **0.0026** |
| **IgG1 Bisection** | -0.11 | 0.61 |

| | | |
|---|---|---|
| **IgG1 Fucosylation** | 0.14 | 0.54 |
| **IgG1 Galactosylation** | 0.094 | 0.68 |
| **IgG1 Sialylation** | 0.37 | 0.091 |
| **IgG1 Sialylation per galactose** | 0.44 | 0.04 |
| **IgG2/3 Hybrid-type** | 0.61 | **0.0024** |
| **IgG2/3 Bisection** | 0.15 | 0.51 |
| **IgG2/3 Fucosylation** | 0.19 | 0.39 |
| **IgG2/3 Galactosylation** | -0.093 | 0.68 |
| **IgG2/3 Sialylation** | 0.036 | 0.87 |
| **IgG2/3 Sialylation per galactose** | 0.23 | 0.3 |
| **IgG4 Bisection** | 0.13 | 0.58 |
| **IgG4 Galactosylation** | -0.26 | 0.26 |
| **IgG4 Sialylation** | 0.079 | 0.73 |
| **IgG4 Sialylation per galactose** | 0.33 | 0.14 |

| | **PCT in meningococcal patients (4 to 18 years old)** | |
|---|---|---|
| | **Spearman's correlation coefficient (r)** | ***p*-value** |
| **IgG1 Hybrid-type** | 0.11 | 0.7 |
| **IgG1 Bisection** | -0.38 | 0.18 |
| **IgG1 Fucosylation** | 0.38 | 0.18 |
| **IgG1 Galactosylation** | 0.24 | 0.41 |
| **IgG1 Sialylation** | -0.0066 | 0.99 |
| **IgG1 Sialylation per galactose** | -0.19 | 0.52 |
| **IgG2/3 Hybrid-type** | -0.029 | 0.93 |
| **IgG2/3 Bisection** | -0.26 | 0.37 |
| **IgG2/3 Fucosylation** | 0.011 | 0.98 |
| **IgG2/3 Galactosylation** | 0.059 | 0.84 |
| **IgG2/3 Sialylation** | 0.09 | 0.76 |
| **IgG2/3 Sialylation per galactose** | -0.099 | 0.74 |
| **IgG4 Bisection** | -0.011 | 0.98 |
| **IgG4 Galactosylation** | -0.25 | 0.41 |
| **IgG4 Sialylation** | -0.21 | 0.49 |
| **IgG4 Sialylation per galactose** | -0.099 | 0.75 |

| | **TNF in meningococcal patients (4 to 18 years old)** | |
|---|---|---|
| | **Spearman's correlation coefficient (r)** | ***p*-value** |
| **IgG1 Hybrid-type** | -0.14 | 0.59 |
| **IgG1 Bisection** | -0.28 | 0.28 |
| **IgG1 Fucosylation** | 0.019 | 0.94 |
| **IgG1 Galactosylation** | 0.052 | 0.84 |
| **IgG1 Sialylation** | -0.21 | 0.41 |
| **IgG1 Sialylation per galactose** | -0.25 | 0.33 |
| **IgG2/3 Hybrid-type** | -0.2 | 0.43 |
| **IgG2/3 Bisection** | -0.22 | 0.4 |
| **IgG2/3 Fucosylation** | -0.32 | 0.21 |
| **IgG2/3 Galactosylation** | 0.045 | 0.86 |
| **IgG2/3 Sialylation** | -0.041 | 0.87 |
| **IgG2/3 Sialylation per galactose** | -0.23 | 0.38 |
| **IgG4 Bisection** | -0.15 | 0.57 |
| **IgG4 Galactosylation** | 0.14 | 0.59 |
| **IgG4 Sialylation** | -0.3 | 0.26 |
| **IgG4 Sialylation per galactose** | -0.4 | 0.13 |

| | IL6 in meningococcal patients (4 to 18 years old) | |
| --- | --- | --- |
| | Spearman's correlation coefficient (r) | *p* -value |
| **IgG1 Hybrid-type** | -0.19 | 0.51 |
| **IgG1 Bisection** | -0.69 | 0.0058 |
| **IgG1 Fucosylation** | 0.35 | 0.21 |
| **IgG1 Galactosylation** | 0.28 | 0.31 |
| **IgG1 Sialylation** | -0.021 | 0.94 |
| **IgG1 Sialylation per galactose** | -0.22 | 0.43 |
| **IgG2/3 Hybrid-type** | -0.13 | 0.64 |
| **IgG2/3 Bisection** | -0.45 | 0.092 |
| **IgG2/3 Fucosylation** | -0.068 | 0.81 |
| **IgG2/3 Galactosylation** | 0.34 | 0.22 |
| **IgG2/3 Sialylation** | 0.24 | 0.39 |
| **IgG2/3 Sialylation per galactose** | -0.089 | 0.75 |
| **IgG4 Bisection** | -0.48 | 0.087 |
| **IgG4 Galactosylation** | 0.081 | 0.78 |
| **IgG4 Sialylation** | -0.12 | 0.69 |
| **IgG4 Sialylation per galactose** | -0.18 | 0.54 |

| | IL8 in meningococcal patients (4 to 18 years old) | |
| --- | --- | --- |
| | Spearman's correlation coefficient (r) | *p* -value |
| **IgG1 Hybrid-type** | -0.28 | 0.31 |
| **IgG1 Bisection** | -0.62 | 0.016 |
| **IgG1 Fucosylation** | 0.13 | 0.64 |
| **IgG1 Galactosylation** | 0.16 | 0.57 |
| **IgG1 Sialylation** | -0.1 | 0.71 |
| **IgG1 Sialylation per galactose** | -0.24 | 0.39 |
| **IgG2/3 Hybrid-type** | -0.13 | 0.64 |
| **IgG2/3 Bisection** | -0.35 | 0.2 |
| **IgG2/3 Fucosylation** | -0.26 | 0.35 |
| **IgG2/3 Galactosylation** | 0.2 | 0.47 |
| **IgG2/3 Sialylation** | 0.12 | 0.66 |
| **IgG2/3 Sialylation per galactose** | -0.17 | 0.55 |
| **IgG4 Bisection** | -0.59 | 0.03 |
| **IgG4 Galactosylation** | 0.09 | 0.76 |
| **IgG4 Sialylation** | -0.15 | 0.62 |
| **IgG4 Sialylation per galactose** | -0.27 | 0.35 |
