# Supplementary material for: Differences in IgG Fc Glycosylation Are Associated with Outcome of Pediatric Meningococcal Sepsis
Source: mBio. 2018 Jun 19;9(3):e00546-18. doi: 10.1128/mBio.00546-18 (PMC6016251; doi:10.1128/mBio.00546-18)
Supplement: TEXT S1 [file mbo003183922s1.pdf]

Text S1. Supplementary Materials and Methods

NanoLC-MS(/MS) analysis of glycopeptides

The IgG digest was separated and analyzed by an Ultimate 3000 HPLC system (Dionex Corporation, Sunnyvale, CA), coupled to a Maxis Impact HD quadrupole time-of-flight mass spectrometer (q-TOF-MS; Bruker Daltonics) as described before (1). The HPLC system consisted of a gradient pump, an isocratic loading pump, an autosampler maintained at 4 °C and a column oven maintained at 30 °C. Of each sample 250 nL was injected into a flow of 25 µL/min of solvent A (aqueous 0.1 % TFA (*v/v*)) and trapped on the trap column (Dionex Acclaim PepMap100 C18, 5 mm × 300 µm; Thermo Fisher Scientific, Breda, The Netherlands). With a flowrate of 900 nL/min the analytes were eluted on a C18 nano-column (Ascentis Express C18 nanoLC column, 50 mm × 75 µm, 2.7 µm fused core particles; Supelco, Bellefonte, PA) and separated in a linear gradient from 3% to 30% solvent B (95% ACN (*v/v*)) in 5 min. The interface between the HPLC system and the q-TOF-MS was equipped with CaptiveSpray and nanoBooster technologies (Bruker Daltonics), using ACN-doped nebulizing gas (pressure: 0.2 bar; dry gas flow: 3.0 L/min; dry temperature: 180 °C). Profile spectra were recorded in *m/z* range 550 to 1800 with a frequency of 1 Hz. The collision energy was 7.0 eV, the transfer time 110 µs, and the pre-pulse storage 21 µs. The total analysis time per sample was 12 min. The HPLC system and the q-TOF-MS were operated under Chromeleon Client v6.80 build 3161 and otofControl v3.4 build 14, respectively.

Data processing

The raw LC-MS data were extracted and curated using LacyTools v0.0.7.2 as described previously (1, 2). Briefly, the chromatograms of each run were aligned based on the exact mass and the average retention time over all runs of the three most abundant glycoforms of each IgG subclass; H3N4F1, H4N4F1 and H5N4F1 (H: Hexose, N: *N*-acetylhexosamine, F: Fucose). An alignment time window of ± 10 s and a mass window of ± 0.1 Th were used. Glycopeptide peaks were used for alignment only when their signal-to-noise ratio (S/N) was above nine, with a minimum of seven glycopeptides per sample. Using the described separation methods, glycopeptides with the same peptide portion co-eluted. This resulted in three glycopeptide clusters: one for IgG1, one for IgG4 and one for the combination of IgG2 and 3. The tryptic Fc glycopeptides for IgG2 and 3 have identical peptide moieties in the Caucasian population and are therefore not distinguishable by this profiling method. After alignment, sum spectra were created per glycopeptide cluster, with a time window of ± 12 s per cluster. Sum spectra were calibrated based on at least five glycopeptides per cluster with a S/N higher than nine; for all IgG subclasses H3N4F1, H4N4F1, H5N4F1, H5N5F1 and H5N4F1S1 were used for calibration. The mass window used for calibration was 0.3 Da. For the targeted extraction, the

areas of at least 95% of the isotopic envelope were integrated for the initial analytes, specified based on manual annotation of summed spectra per biological class (healthy or meningococcal sepsis), both as doubly charged and as triply charged species. Background subtraction was performed based on local background calculations. Spectra were excluded from further analysis when the total spectrum intensity was below ten times the average spectrum intensity of the blanks. In this way, no spectra were excluded for IgG1, 15 spectra were excluded for IgG2/3 and 29 spectra were excluded for IgG4. Analytes were included in the final data analysis when their average S/N (calculated per biological class) was above nine, their isotopic pattern did not, on average, deviate more than 20% from the theoretical pattern and their average mass error was within ± 10 ppm. This resulted in the extraction of 22 IgG1, 15 IgG2/3 and 10 IgG4 glycoforms (**Table S2** in Supporting information).

1. Falck D, Jansen BC, de Haan N, Wuhrer M. 2017. High-Throughput Analysis of IgG Fc Glycopeptides by LC-MS. Methods Mol Biol 1503:31-47.
2. Jansen BC, Falck D, de Haan N, Hipgrave Ederveen AL, Razdorov G, Lauc G, Wuhrer M. 2016. LaCyTools: A Targeted Liquid Chromatography-Mass Spectrometry Data Processing Package for Relative Quantitation of Glycopeptides. J Proteome Res 15:2198-210.
